# Multifunctional Adaptive NS1 Mutations Are Selected upon Human Influenza Virus Evolution in the Mouse

**DOI:** 10.1371/journal.pone.0031839

**Published:** 2012-02-21

**Authors:** Nicole E. Forbes, Jihui Ping, Samar K. Dankar, Jian-Jun Jia, Mohammed Selman, Liya Keleta, Yan Zhou, Earl G. Brown

**Affiliations:** 1 Department of Biochemistry, Microbiology and Immunology, Faculty of Medicine, University of Ottawa, Ottawa, Ontario, Canada; 2 Emerging Pathogens Research Centre, Faculty of Medicine, University of Ottawa, Ottawa, Ontario, Canada; 3 Vaccine and Infectious Disease Organization, University of Saskatchewan, Saskatoon, Saskatchewan, Canada; 4 Canadian Institutes of Health Research (CIHR) Canadian Influenza Pathogenesis Team, University of Ottawa, Ottawa, Ontario, Canada; Johns Hopkins University - Bloomberg School of Public Health, United States of America

## Abstract

The role of the NS1 protein in modulating influenza A virulence and host range was assessed by adapting A/Hong Kong/1/1968 (H3N2) (HK-wt) to increased virulence in the mouse. Sequencing the NS genome segment of mouse-adapted variants revealed 11 mutations in the NS1 gene and 4 in the overlapping NEP gene. Using the HK-wt virus and reverse genetics to incorporate mutant NS gene segments, we demonstrated that all NS1 mutations were adaptive and enhanced virus replication (up to 100 fold) in mouse cells and/or lungs. All but one NS1 mutant was associated with increased virulence measured by survival and weight loss in the mouse. Ten of twelve NS1 mutants significantly enhanced IFN-β antagonism to reduce the level of IFN β production relative to HK-wt in infected mouse lungs at 1 day post infection, where 9 mutants induced viral yields in the lung that were equivalent to or significantly greater than HK-wt (up to 16 fold increase). Eight of 12 NS1 mutants had reduced or lost the ability to bind the 30 kDa cleavage and polyadenylation specificity factor (CPSF30) thus demonstrating a lack of correlation with reduced IFN β production. Mutant NS1 genes resulted in increased viral mRNA transcription (10 of 12 mutants), and protein production (6 of 12 mutants) in mouse cells. Increased transcription activity was demonstrated in the influenza mini-genome assay for 7 of 11 NS1 mutants. Although we have shown gain-of-function properties for all mutant NS genes, the contribution of the NEP mutations to phenotypic changes remains to be assessed. This study demonstrates that NS1 is a multifunctional virulence factor subject to adaptive evolution.

## Introduction

Highly pathogenic avian influenza (HPAI) H5N1 viruses have the ability to infect human hosts and cause fatal disease [1], [2], with 566 reported human cases resulting in 332 deaths [3]. Currently, the genetic determinants of influenza A virus (IAV) host adaptation are largely unknown [4], [5]. Although HPAI H5N1 has not yet acquired the property of human to human transmission, the possibility of a pathogenic H5N1 virus becoming fully adapted to the human host remains a threat to global health. It is thus necessary to understand the genetic determinants of IAV host range and virulence in order to predict the emergence of strains with pandemic potential.

The IAV non-structural protein 1 (NS1) is expressed from the full length transcript of gene segment 8 that overlaps with the spliced nuclear export protein (NS2/NEP) transcript. Several reports have demonstrated that the NS1 protein contributes to influenza virulence and host range in animal models [5]–[9]. NS1 has multiple regulatory roles during IAV infection that ultimately suppress the host interferon (IFN) antiviral responses and promote virus replication [10]. NS1 inhibits IFN production as well as suppresses IFN antiviral effects via numerous host factor interactions. NS1 inhibits IFN production by interfering with the cytoplasmic viral RNA sensor retinoic acid-inducible gene 1 (RIG-I), both by directly binding RIG-I to block activation of IFN mRNA transcription [11]–[13] and by binding the ubiquitin ligase tripartite motif-containing protein 25 (TRIM25) that activates RIG-I antiviral signalling [14]. NS1 also blocks IFN-induced STAT-1 signalling to prevent IFN-stimulated gene transcription [15]. NS1 down regulates host gene expression, including IFN expression, by binding the cleavage and polyadenylation specificity factor 30 F2F3 domain (CPSF30-F2F3) [16]–[18], polyA binding protein nuclear 1 (PABPNI) [19], and the nuclear RNA export factor 1/tip-associated protein (NXF1/TAP) [20], and these interactions result in the inhibition of host mRNA processing and/or export. NS1 also binds dsRNA and the dsRNA-dependent protein kinase R (PKR) to prevent dsRNA-induced activation of the antiviral effectors PKR and 2′-5′oligo(A) synthetase (2′-5′ OAS) [21]–[23].

The NS1 protein regulates viral gene expression at multiple levels including splicing. Although NS1 was found to alter the accumulation of segment 7 spliced mRNAs [24], the role of NS1 in gene segment 8 splicing remains unresolved; some reports have demonstrated the splicing of segment 8 is independent of NS1 function [25], [26], while others have shown NS1 blocks the splicing of its own mRNA [27], [28]. In the context of viral gene transcription, NS1 has been found to associate with virus ribonucleoprotein (RNP) complexes *in vivo* [29], and recently Robb *et al.* demonstrated NS1 directly interacts with the A/WSN/1933 (H1N1) nucleoprotein (NP) *in vitro* [30]. Moreover, a recent study identified a novel interaction between NS1 and cellular protein hnRNP-F involved in viral transcription, where depletion of hnRNP-F increased viral RNA polymerase activity and ultimately viral replication [31]. Several studies have shown that the NS1 protein is required for viral RNA synthesis [23], [32], [33]. Moreover, NS1 interaction with the polymerase complex has been associated with the NS1:CPSF30 complex in H5N1-infected cells [34]. In addition to effects on viral transcription, NS1 also enhances viral mRNA translation by direct interaction with cellular translation initiation factors polyA binding protein 1 (PABPI) and eukaryotic initiation factor 4 GI (eIF4GI), which directs the complex to the viral mRNA 5′ untranslated region (5′UTR) to increase viral protein synthesis [35], [36]. The NS1 protein is considered a major virulence factor by enhancing viral gene expression and down regulating both host gene expression and the IFN-mediated antiviral responses.

Mutations and deletions in the NS1 protein have been associated with increased IAV virulence. Highly pathogenic H5N1 viruses isolated from waterfowl, poultry, and humans in Southeast Asia since 2000 possess a 15-nucleotide deletion from position 263–277 in the NS gene [1], [37], [38], resulting in a deletion in residues 80 to 84 that enhances virulence in both chicken and mice [8]. Genetic analyses of other NS1 genes from highly pathogenic H5N1 isolates, A/Goose/Guangdong/1/96 and A/Duck/Guangxi/27/03, have shown mutations V149A and P42S result in high virulence in chickens and mice, respectively [6], [39].

Although NS1 inhibits and activates many host factors, it is not clear which NS1 functions are the primary modulators for adaptive differences among IAVs. In order to identify genetic determinants for IAV virulence and host adaptation, we and others have employed experimental evolution in a novel host to map genetic mutations arising upon adaptation [40]–[44]. To this end, we adapted the human isolate A/Hong Kong (HK)/1/1968 (H3N2) (HK-wt) to increased virulence in the mouse by serial lung-to-lung passage [5], [7], [42], [45], [46]. Using this approach, all mutant mouse-adapted (MA) genome segments in the virulent A/Hong Kong/1/68-MA (HK-MA) variant demonstrated increased disease severity including the NS1 V23A mutation that decreased MLD_50_ by 6 fold compared to the HK-wt virus [46]. Recently, Dankar *et al.* (2011) reported that NS1 gene mutations F103L and M106I selected upon mouse adaptation were convergent with virulence determinants in the H5N1 NS1 gene from A/HK/156/1997, and that expression of these mutations in the HK-wt NS1 gene, as well as in the NS1 gene of the H5N1 human isolate A/HK/156/1997 and in the H9N2 chicken isolate, A/Ck/Beijing/1/1995 increased virulence in the mouse [7]. In the current study, we further assessed the roles of 11 MA mutations in the NS1 gene of A/Hong Kong/1/68 (H3N2) on host adaptation and virulence, and employed reverse genetics using the rHK-wt backbone to demonstrate their effect on replication and virulence in mouse cells and lungs. We found NS1 mutations selected upon mouse adaptation were multifunctional, where numerous mutations increased virulence in the mouse, replication *in vivo* and *in vitro*, viral gene expression *in vitro*, as well as enhanced properties of IFN antagonism.

## Results

### NS1 gene mutations identified in A/Hong Kong/1/1968 mouse adapted variants

**Figure 1:**
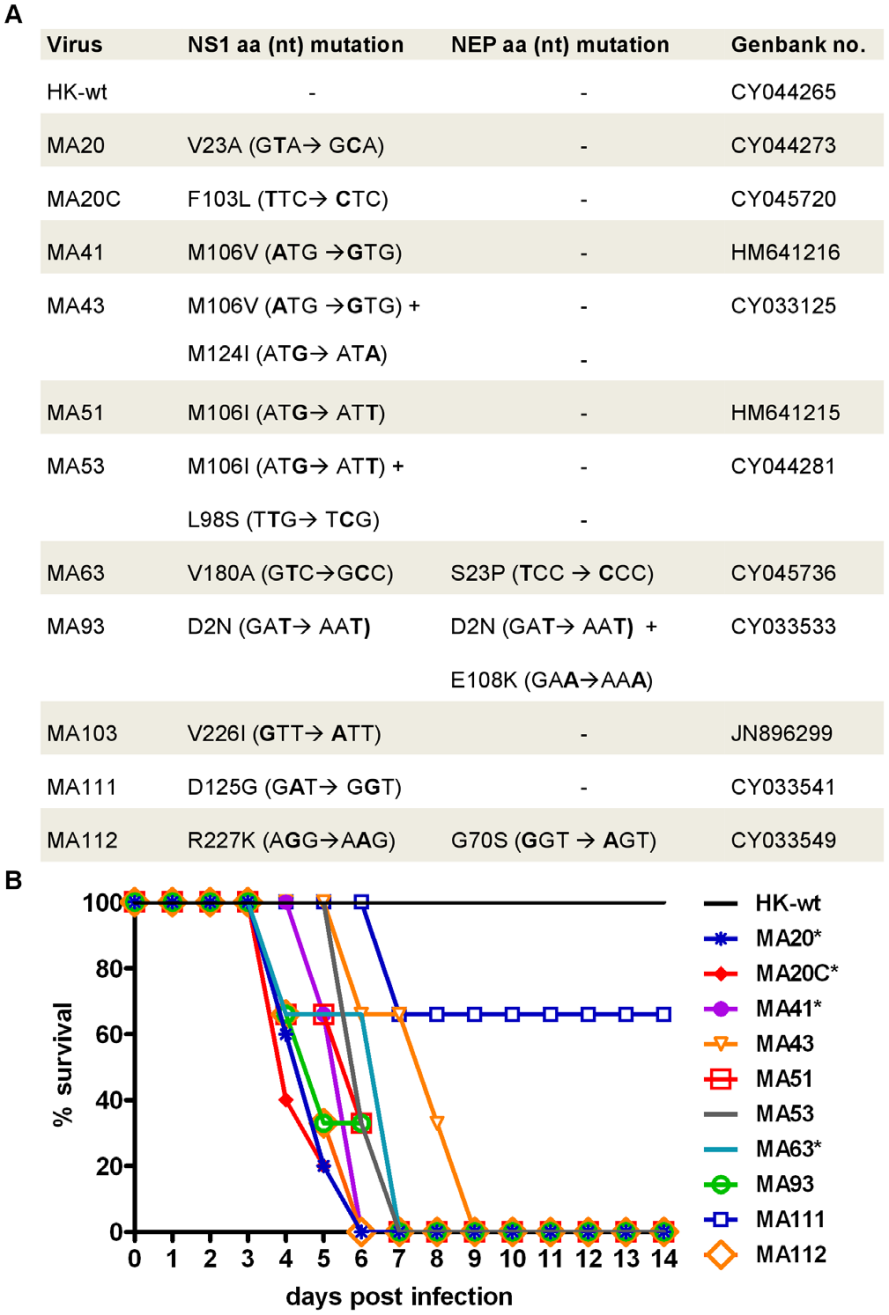
MA viruses with NS mutations demonstrate enhanced virulence in the mouse. (A) Table of MA viruses indicating mutations in the NS gene segment at the protein (aa) and mRNA level (nt) as well as MLD_50_ value in CD-1 mice. (B) Kill curve of MA viruses with NS mutations. CD-1 mice were infected with 1×10^7^ PFU dose (1×10^6^ PFU dose used for viruses indicated with an *) and survival was monitered for 14 days.

We had previously described the experimental evolution of A/Hong Kong/1/1968 (HK-wt) by serial passage in the mouse lung to select highly virulent variants [5], [42], [45], [46]. The genomes of each mouse adapted (MA) variant were previously subjected to full genome sequencing where each variant was found to possess mutations in several viral genes [5]. In respect to the NS1 gene, mouse adapted variants had acquired 11 distinct combinations of single or double mutations within the NS1 gene, 3 of which induced mutations in the overlapping NEP gene (Fig. 1a) [5]. The amino acid substitutions in NS1 and NEP are reproduced here in Figure 1a for representative NS1 mutant MA variants, in addition to the nucleotide substitutions and Genbank accession numbers for each genome segment. We had previously reported that all of these MA mutants (except MA111 (MLD_50_>10^7.5^ PFU) and MA103 (unavailable)) had decreased MLD_50_ values that ranged from 10^1.1^ to 10^6.5^ PFU (geometric mean = 10^4.1^ PFU) relative to the non-virulent parental HK-wt that possessed an MLD_50_>10^7.7^ PFU [5]. Figure 1b shows the survival response of groups of mice infected with 10^7^ or 10^6^ PFU of the indicated MA viruses relative to HK-wt mice to demonstrate the increased virulence of these MA variants that were associated with 100% mortality in all cases except for MA111.

### Generation of recombinant NS mutant Hong Kong viruses

To determine the role of each mutant MA NS1 gene in virulence, recombinant HK viruses were generated to express the mutations of each mouse adapted NS gene segment (rHK NS mutants). In addition, we generated the NS1 mutant L98S, a mutation that was selected in combination with the M106I mutation upon mouse adaption (MA53, Fig. 1a). rHK NS mutants with NEP mutations (Fig. 1a) were indicated by an ^†^ throughout the text. The NS gene segments of all rescued viruses were sequenced to verify that they were free of unwanted mutations. All rHK viruses grew to high titre (>1×10^7^ PFU/mL) in the allantoic cavity of SPF chicken embryos as determined by plaque assay on MDCK cell monolayers.

### NS1 mutations selected upon mouse adaption enhance virulence in mice

**Figure 2:**
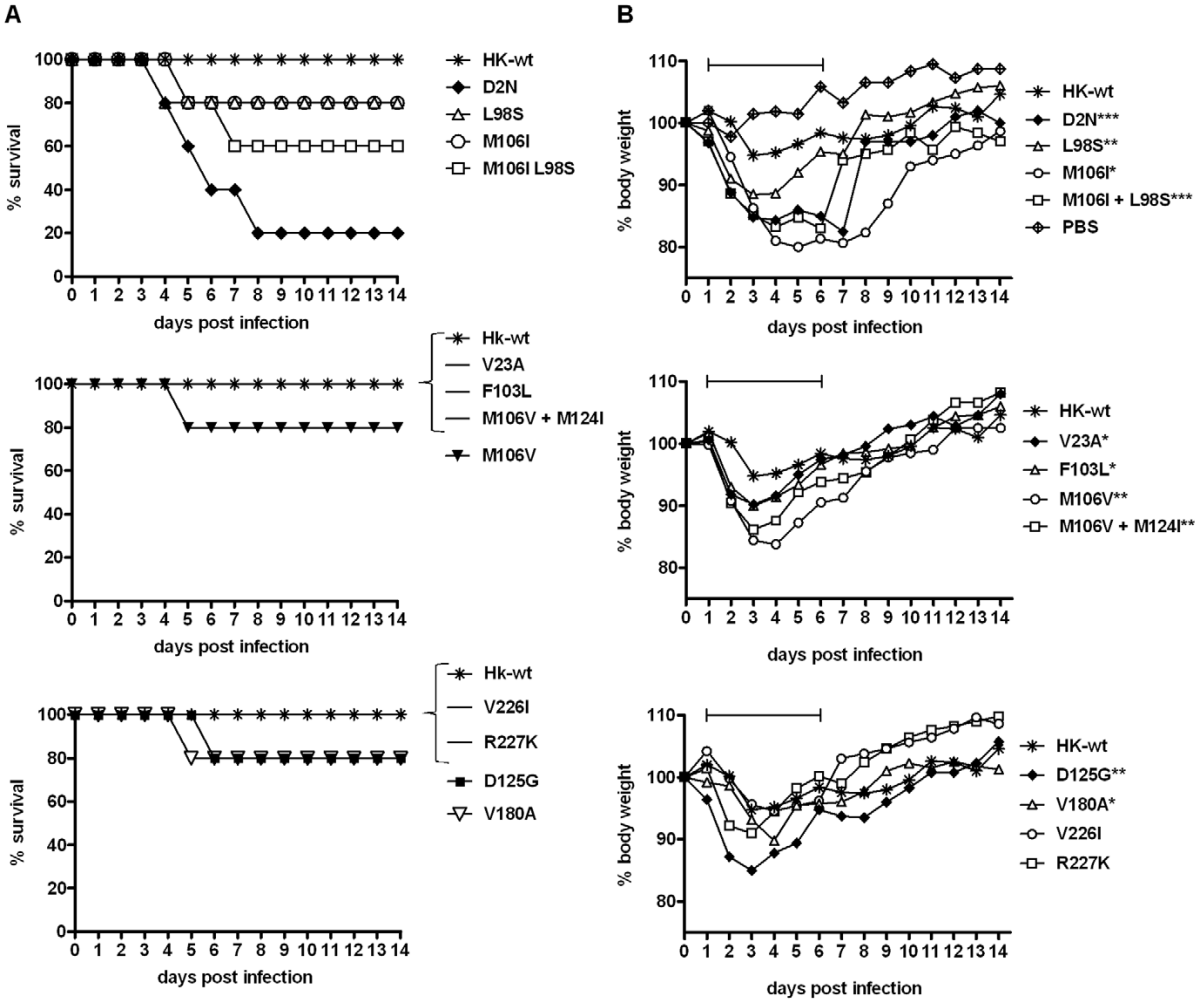
Mouse adapted NS1 mutations increase virulence in CD-1 mice. Groups of 5 female CD-1 mice were infected intranasally with 5×10^6^ PFU dose of rHK NS MA or HK-wt virus. 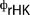 NS1 mutant M106V + M124I was inoculated at dose of 2.5×10^6^ PFU due to insufficient viral stock titre. Survival (A) and body weight loss (B) were monitored for 14 dpi. Percent body weight is expressed as the mean value of 5 (or total number alive) mice (*p<0.05, **p<0.01, ***p<0.001; two-tailed unequal variance paired student's t-test for days 1–6). Experimental endpoint was defined as >30% body weight loss and respiratory distress. Graphical analysis of data was broken into three mutant sets, with rHK-wt included in each, due to the number of mutants surveyed.

To assess the effect of NS1 mutations on virulence in mice, groups of 5 or 6 CD-1 mice were infected intranasally with rHK-wt as well as rHK NS mutants. High dose infection with rHK-wt (5×10^6^ PFU) in mice did not result in mortality (Fig. 2a). In contrast, 7 of the 12 NS1 mutants (D2N^†^, L98S, M106V, M106I, M106I+L98S, D125G, and V180A^†^) resulted in 20% to 83% mortality in the mouse (Fig. 2a) at the same dosage of infection. The virus possessing the NS1 mutation D2N^†^ was the most virulent, causing 83% mortality (5 of 6 mice), and resulted in a >30 fold reduction of MLD_50_ value compared to the rHK-wt virus (10^6.2^ PFU versus>10^7.7^ PFU). The NS1 M106I+L98S mutant caused 40% mortality (2 of 5 mice), whereas the single mutants of this combination, M106I and L98S, as well as mutants M106V, D125G, and V180A^†^, all caused 20% mortality (Fig. 2a). Although several NS1 mutants did not increase mortality, the majority of MA NS1 mutations increased disease severity, as measured by body weight loss. Mouse body weight loss following infection of the rHK-wt virus reached a maximum average loss of 5%, whereas all mutants induced significantly more weight loss than rHK-wt from days 1–6 post infection, (p-values range from <0.05 to <0.001; two-tailed paired, t-test), with the exception of NS1 mutants V226I and R227K^†^ that did not result in statistically significant decreased body weight loss (Fig. 2b). The extent of body weight loss in general correlated with mortality however the M106I mutant virus induced the greatest weight loss but resulted in less mortality than the two most virulent mutants, D2N^†^ and M106I+L98S, which both induced the next greatest statistically significant increase in body weight loss compared to rHK-wt (p<0.001, two tailed, paired, t-test). In addition, all the mutants causing fatality of 1 of 5 mice (L98S, M106I, M106V, D125G, and V180A^†^) also demonstrated a significant decrease in body weight compared to rHK-wt from days 1–6 pi (p<0.01, p<0.05, p<0.01, p<0.01, and p<0.05, two-tailed, paired, t-test) (Fig. 2b). These observations show that the majority of single and double NS1 mutations acquired upon adaptation to the mouse possessed properties of increased virulence.

### NS1 mutants D2N^†^, M106I, L98S and M106I+L98S increase virus infection and pathology in the mouse lung

**Figure 3:**
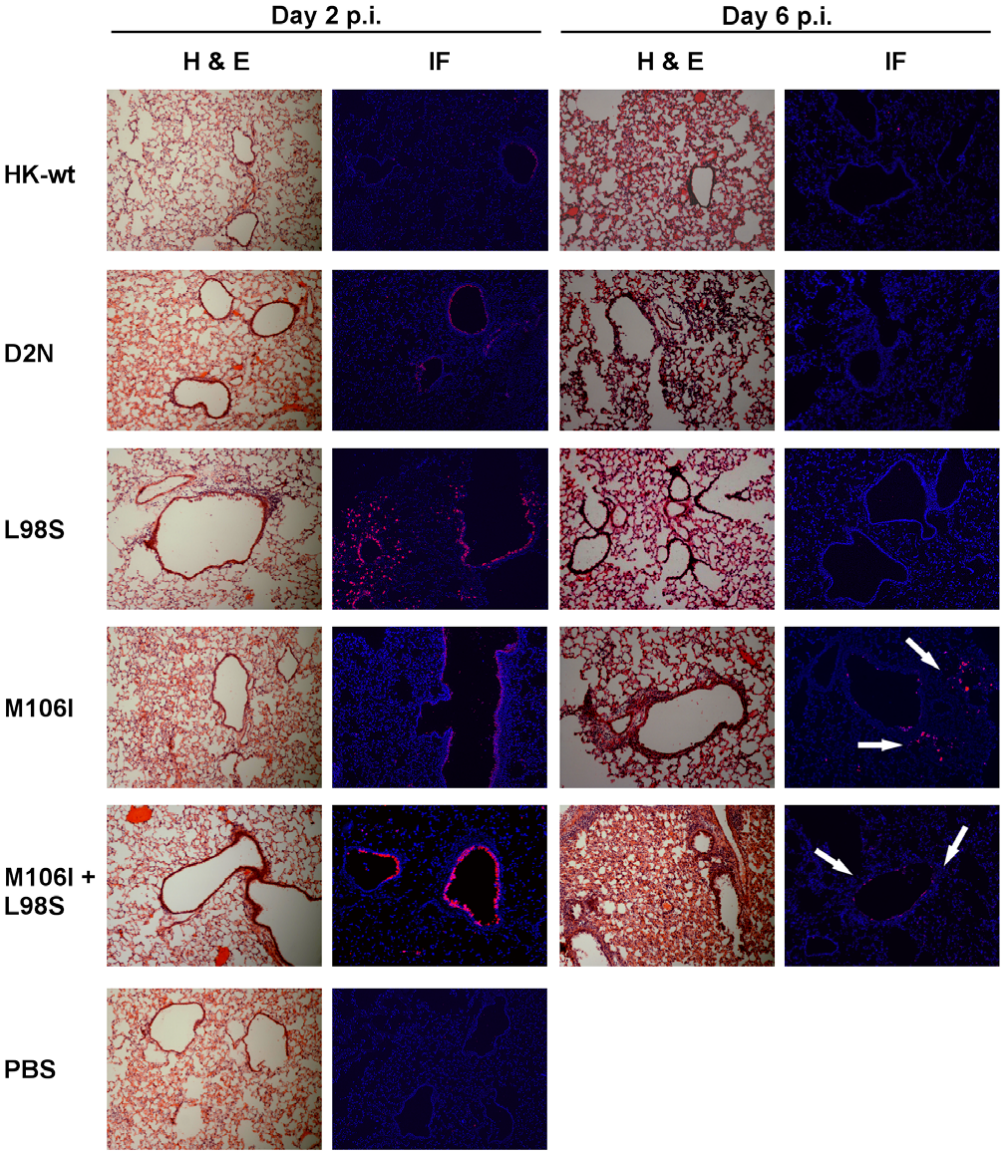
Mouse adapted NS1 mutations increase pathology and virus spread in the mouse lung. Groups of 4 CD-1 mice were infected with 1×10^5^ PFU dose of selected rHK NS mutant or rHK-wt viruses and two sets of lungs were collected at both 2 and 6 dpi. IF: Virus was detected by immunofluorescence; frozen lung sections were stained with anti-HK primary antibody, Cy3- conjugated secondary antibody (red), and nuclei were stained with Hoeschst (blue). Images were taken using a 20× objective lens. White arrows indicate foci of staining at 6 dpi. H&E: Lung pathology was assessed by H&E staining. Images were taken using a 10× objective lens.

Given that NS1 mutants D2N^†^ and M106I+L98S induced the greatest mortality in the mouse (Fig. 2a), we assessed these combinations of mutations as well as the single mutants L98S and M106I for their role in lung tropism and lung pathology following intranasal infection using a dose of 1×10^5^ PFU/mouse. Pathology and extent of lung tissue infection was determined by H&E and immunofluorescent staining of HK viral antigen, respectively, on frozen lung sections collected 2 and 6 dpi. The rHK-wt virus antigen was detected in focal regions in the bronchi at day 2 but not day 6 pi, however at day 2 the NS1 mutants D2N^†^, M106I, L98S and M106I+L98S all infected bronchiolar regions to a greater extent (Fig. 3). In addition, mutants D2N^†^, L98S and M106I+L98S increased virus spread to alveolar regions of the lung (Fig. 3). At day 6 pi, viral antigens persisted in the mouse lung in both alveolar and bronchiole regions for NS1 mutants M106I and M106I+L98S (Fig. 3, white arrows). Lung pathology was mild for rHK-wt, where H&E staining was similar to mock-infected (PBS) lungs at day 2 pi, and moderate congestion with increased inflammatory cell recruitment was seen for the bronchiole region at day 6 pi (Fig. 3). Increased lung pathology including alveolar thickening and cellular recruitment was observed at 2 dpi for NS1 mutants L98S and M106I+L98S, and at 6 dpi for NS1 mutants D2N^†^, M106I, and M106I+L98S (Fig. 3). The enhanced pathology and spread within the lung demonstrated increased abilities to overcome host resistance due these NS1 mutations, which in turn resulted in greater virus replication and disease severity.

### Mouse-adapted NS1 mutations limit IFN β production and enhance virus yield in the mouse lung

**Figure 4:**
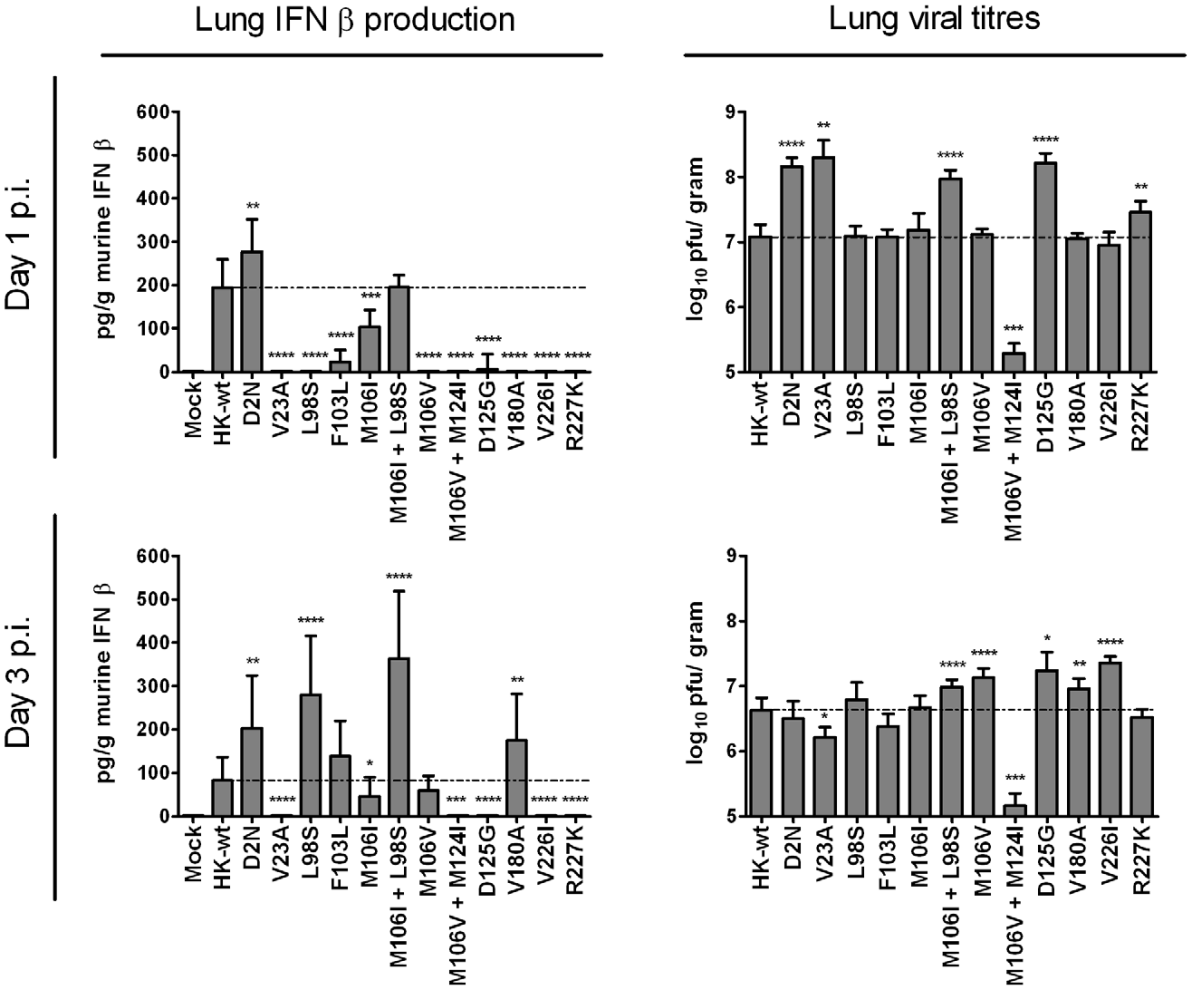
Mouse-adapted NS1 mutations affect IFN-β production and viral yield in the mouse lung. Groups of 6–12 female CD-1 mice were infected intranasally with 1×10^5^ PFU dose of rHK NS mutants or rHK-wt or mock-infected with PBS. Lungs were collected 1 and 3 days pi, homogenized, and quantified for IFN β concentration using a commercial murine IFN β ELISA kit (left panel), and for viral titre by plaque assay on MDCK cells (right panel) Data represent the means ± SD (*p<0.05, **p<0.01, ***p<0.001, ****p<0.0001; two-tailed unequal variance student's t-test).

We have demonstrated that the majority of NS1 mutations selected upon mouse adaptation increased virulence in CD-1 mice. To assess the effect of MA NS1 mutations on IFN-β production, IFN β levels were determined for infected mouse-lung extracts. We infected groups of 3–6 mice intranasally with rHK-wt and rHK NS mutants (1×10^5^ PFU dose) and then measured IFN-β concentration in the lung extracts at both 1 and 3 days post-infection using ELISA with subtraction of values from mock PBS infected lungs. rHK-wt induced an average of 200 pg/g IFN-β in the mouse lung 1 day following infection and all of the NS1 mutants induced significantly less IFN-β except mutants D2N^†^ and M106I+L98S (Fig. 4, upper left panel). NS1 mutant D2N^†^ was the sole mutant to induce significantly more lung IFN-β production than rHK at 1 dpi (p<0.01). Notably, numerous NS1 mutants restricted the amount of IFN-β produced to below the limit of detection 1 dpi (V23A, L98S, M106V, M106V+M124I, V180A^†^, V226I, and R227K^†^) (p<0.0001) (Fig. 4, upper left panel). IFN-β levels in lungs collected 3 days pi were significantly higher than rHK-wt for NS1 mutants D2N^†^, L98S, M106I+L98S, and V180A^†^ (p<0.01, p<0.0001, p<0.0001, p<0.01, respectively). However the amount of IFN-β produced was below the limit of detection for five NS1 mutants at 3 dpi, mutants V23A, M106V+M124I, D125G, V226I, and R227K^†^.

Given that IFN-β induction is dependent on the extent of virus replication, we also determined the viral yield of each lung sample used to determine IFN-β concentration. The rHK-wt virus grew to 1.2×10^7^ PFU/gram 1 day pi, and then decreased to 4.3×10^6^ PFU/g at day 3 pi (Fig. 4, right panels). All mutants except L98S, F103L, M106I, and M106V + M124I resulted in enhanced yields at one or both time points. Enhanced lung viral titre was maintained at both day 1 and day 3 pi for NS1 mutants M106I + L98S and D125G. At day 1 pi, NS1 mutants D2N^†^, V23A, and D125G grew to titres that exceeded rHK-wt >10 fold (p<0.0001, p<0.01, p<0.0001, respectively) and NS1 mutants M106I+L98S and R227K^†^ also significantly exceeded rHK-wt titres (p<0.0001, p<0.01, respectively) (Fig. 4, upper right panel). At day 3 pi, lung titres for NS1 mutants M106I+L98S, M106V, D125G, V180A^†^, and V226I were significantly greater than rHK-wt (p<0.0001, p<0.0001, p<0.05, p<0.01, p<0.0001, respectively) (Fig. 4, lower right panel). Compared to rHK-wt titre, viral growth was significantly attenuated for NS1 mutant M106V + M124I at both day 1 and 3 pi (p<0.001), as well as NS1 mutant V23A at day 3 pi (p<0.05) (Fig. 4, right panel).

The reduced production of IFN-β that was associated with similar or significantly higher viral replication therefore indicated that these mutations function to reduce IFN-β production. The reduced replication seen for NS1 mutant M106V + M124I may account for the reduction in IFN-β produced by these mutants, however role of the M124I mutation in this combination is unknown. We thus conclude that NS1 mutants selected upon mouse adaptation, with the exception of mutant M106V + M124I, are capable of reducing the amount of IFN-β produced in the mouse lung while maintaining or enhancing the level of viral replication.

### Identification of NS1 mutations that affect CPSF30-F2F3 binding

**Figure 5:**
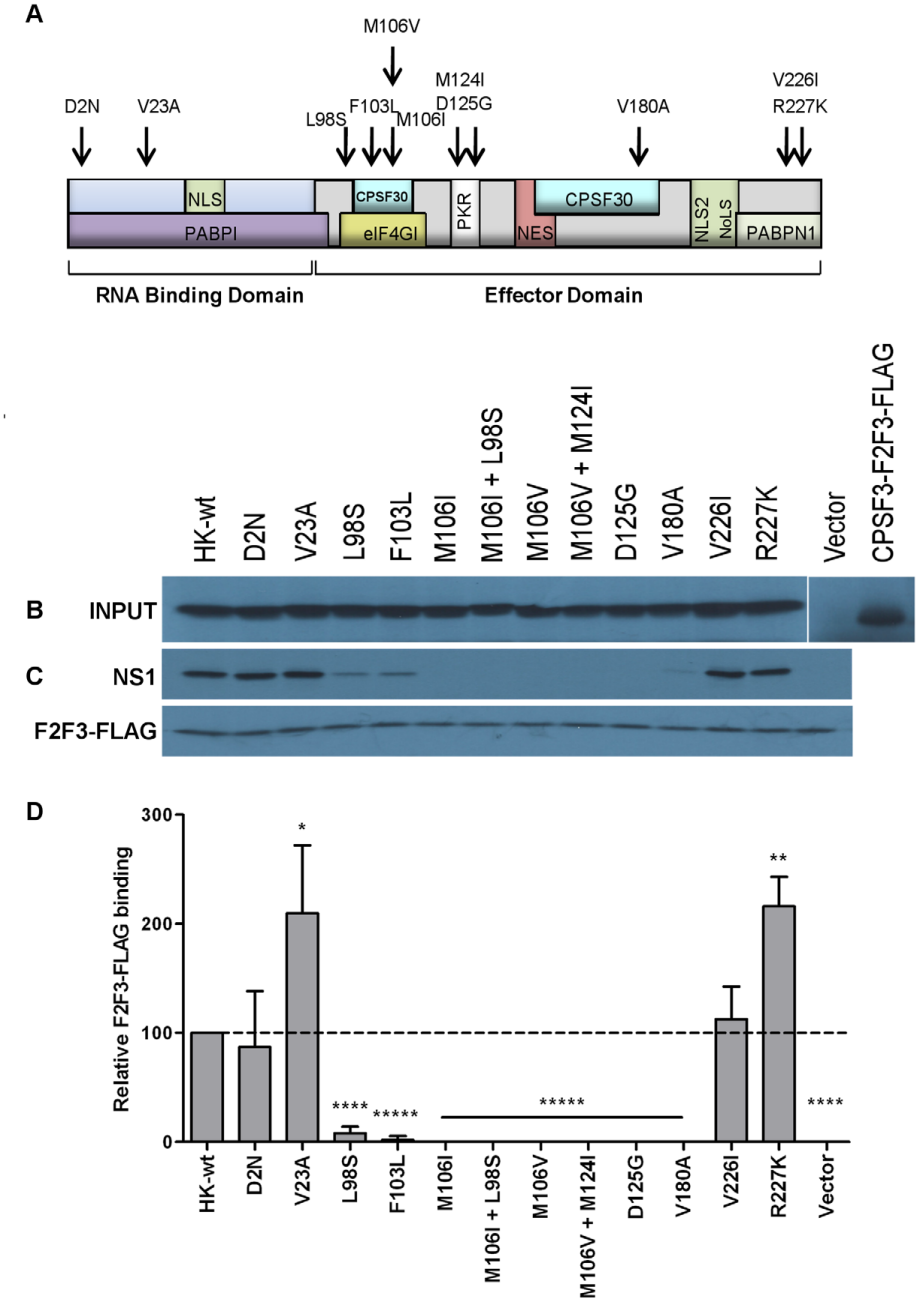
Mouse-adapted NS1 mutants affect binding affinity to CPSF30-F2F3. (A) Map of NS1 protein indicating the location of NS1 mutations selected upon adaption of A/Hong Kong/1/1968 (H3N2) to the mouse, as depicted by black arrows. NS1 binding domains are depicted within and below the protein diagram. (B) NS1 proteins (wt or mutant) as well as CPSF30-F2F3-FLAG were expressed by *in vitro* coupled transcription and translation of expression plasmids or empty vector in the presence of ^35^S labeled methionine and cysteine and expression was evaluated by autoradiography. Radiolabeled mutant or HK-wt NS1 proteins or empty vector were mixed with radiolabeled CPSF30-F2F3-FLAG and subject to pull-down using α-FLAG M2 antibody bound to Protein G Dynabeads then SDS-PAGE electrophoresis and autoradiography. (C) Representative pull-down of 3 independent replicates. (D) Pull-down SDS gels were subject to PhosphorScreen signal amplification, then band intensity quantification using GE Imagequant software. Data represent the means ± SD (*p<0.05, **p<0.01, *** p<0.001, ****p<0.0001; *****p<0.00001; two-tailed student's t-test) for relative NS1 binding.

The majority of MA NS1 mutations mapped within binding domains for cellular binding partners involved in mRNA translation (eIF4GI and PABPI), post transcriptional processing (CPSF30 and PAPBN1) and the host antiviral response (PKR) (Fig. 5a). Given that previous studies have identified a role for NS1: CPSF30 interaction in interferon antagonism [18], [34], but that other reports have associated CPSF30 binding with general inhibition of host gene expression but not as a requirement for inhibition of IFN-β [16], [47], [48], we next determined whether any of the NS1 mutations selected upon mouse adaptation affected NS1 binding to the F2F3 domain of human CPSF30, which is identical in sequence to the mouse CPSF F2F3 domain [5]. Previously we had shown that recombinant 6xHis-tagged NS1 proteins with MA mutations at positions 103, 106 and 180 but not 23 or HK-WT had lost the ability to bind CPSF [5]. We next assessed CPSF binding of the untagged NS1 mutants listed in Figure 1a as well as NS1 mutant L98S. Briefly, mutant and HK-wt NS gene segments as well as FLAG-tagged CPSF-F2F3 (FLAG-F2F3) fragment were expressed *in vitro* by coupled transcription and translation in the presence of ^35^S-methionine and cysteine with detection by autoradiography following SDS-PAGE (Fig. 5b). In an anti-FLAG antibody pulldown assay conducted in triplicate, ^35^S-labeled HK-wt and mutant NS proteins were mixed with FLAG-F2F3 and precipitated using anti-FLAG M2 antibody and Protein G Dynabeads (Invitrogen). Bound proteins were separated by SDS-PAGE and detected by autoradiography (Fig. 5c) and quantified by phosphorscreen detection (Fig. 5d). To verify specific binding to the anti-FLAG antibody, a control pull-down was performed in the absence of F2F3-FLAG protein, which showed a lack of non-specific binding for all NS1 mutants (Fig. S1).

The NS1 mutants D2N^†^ and V226I bound F2F3-FLAG with comparable affinity to HK-wt NS1, but NS1 mutants V23A and R227K^†^ demonstrated a 2-fold increase in binding affinity (p<0.05 and p<0.01, two-tailed student's t-test). However, the majority of NS1 mutants either partially or fully suppressed NS1-F2F3 binding. NS1 mutants L98S and F103L bound at detectable but significantly decreased levels than HK-wt NS1 (p<0.0001, p<0.00001) (Fig. 5d). NS1 mutants M106I, M106I + L98S, M106V, M106V + M124I, D125G, and V180A^†^ demonstrated undetectable binding to F2F3 in any of the three independently conducted anti-FLAG F2F3 pulldowns (p<0.00001, Fig. 5d). The data indicate that altered NS1:CPSF30-F2F3 binding affinity is a common property for NS1 mutations selected upon mouse adaptation, where two mutants demonstrated enhanced affinity and the majority demonstrated a marked decrease in binding affinity. These results show a lack of correlation between IFN-β induction and CPSF binding.

### Mouse-adapted NS1 mutations enhance viral growth *in vitro*

**Figure 6:**
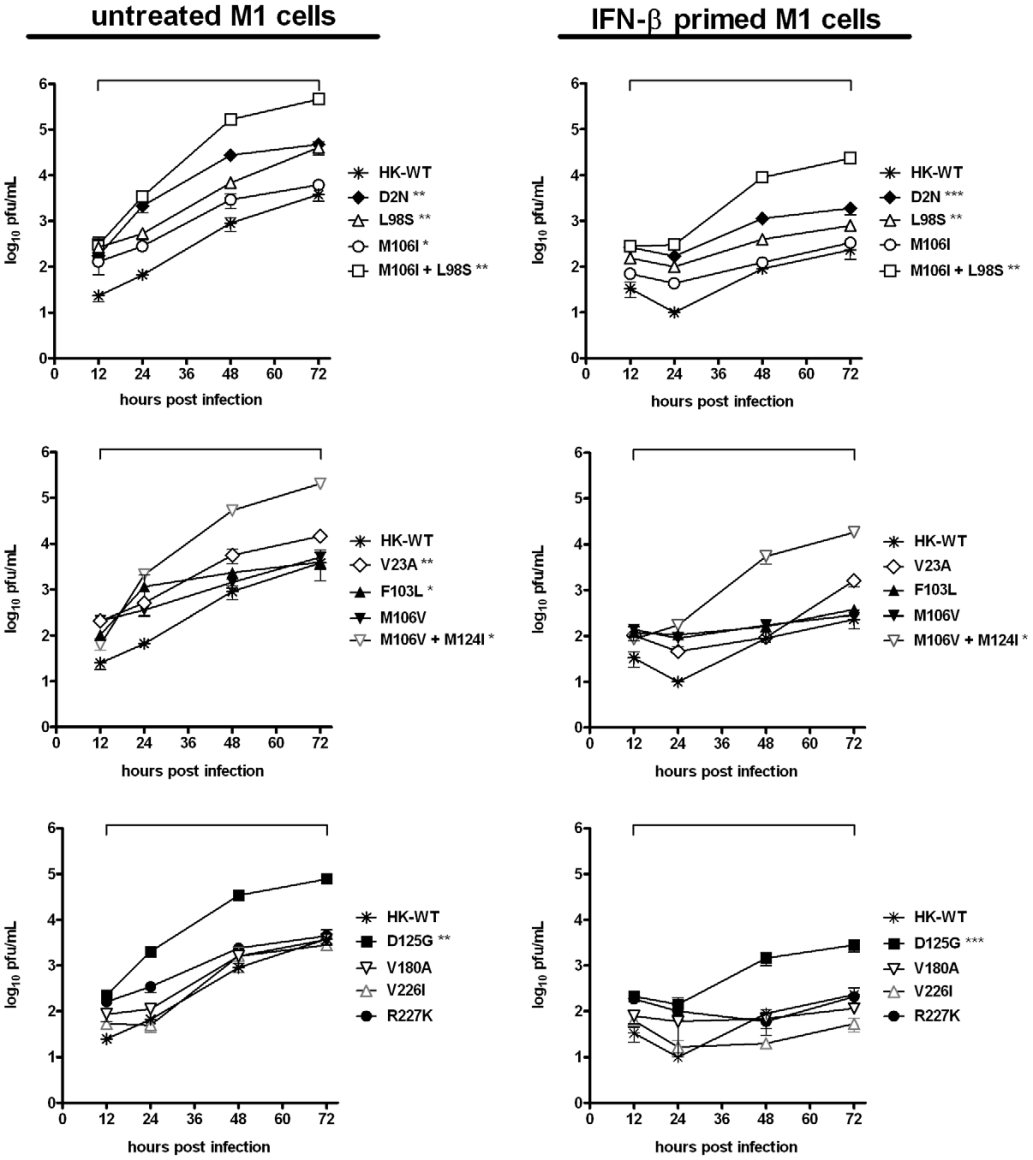
Mouse-adapted NS1 mutations enhance virus growth in untreated and in IFN-β primed mouse cells *in vitro*. Mouse M1 cells were untreated (left panel) or pre-treated with mouse IFN-β (1000 U/mL) for 24 hours (right panel), then infected in triplicate at an MOI of 0.02 with rHK NS mutants or rHK-wt virus, and supernatant collected 12, 24, 48, and 72 hpi was quantified for viral titre by plaque assay on MDCK cells. Graphical analysis of data was broken into three mutant sets, with rHK-wt included in each, due to the number of mutants surveyed. Data represent the means ± SD (*p<0.05, **p<0.01, ***p<0.001; two-tailed student's paired t-test for 12–72 hpi, indicated by horizontal bracket above time range).

To assess whether the adaptive properties associated with MA NS1 mutants *in vivo* also correlated with an *in vitro* phenotype, we determined the effect of MA NS1 mutations on viral growth in M1 cells, a mouse kidney epithelium cell line with intact IFN production and response pathways [7]. Confluent monolayers were infected with rHK-wt or rHK NS mutants at a low multiplicity of infection (MOI = 0.02) and viral titres were quantified at 12, 24, 48, and 72 hours post infection (hpi). NS1 mutants D2N^†^, V23A, L98S, M106I, M106I+L98S, F103L, M106V + M124I, and D125G all significantly enhanced viral yield throughout the course of infection compared to rHK-wt (p<0.05 by two–tailed, paired, t test) (Fig. 6, left panel). The NS1 double mutant M106I + L98S induced the highest level of virus growth; where at both 48 and 72 hpi the mutant grew to >100 fold higher titre than rHK-wt (p<0.0001 and p<0.001, respectively, by two-tailed single sample t-test). NS1 mutants M106V and R227K^†^ significantly enhanced viral growth at early times (12–24 hpi) compared to rHK-wt (p<0.01 and p<0.05, respectively; two–tailed paired t-test), however their yields were similar to rHK-wt at 72 hpi (Fig. 6, left panel). Mutations V180A^†^ and V226I resulted in viral growth curves that were not significantly different from rHK-wt (Fig. 6, left panel). All assayed recombinants reached maximum virus yield at 72 hpi; this titre was significantly increased for NS1 mutants D2N^†^, V23A, L98S, M106I+L98S, M106V + M124I, and D125G compared to rHK-wt (p<0.05 by single sample t-test); however, the contributions of the NS1 mutations in enhancing viral fitness is unknown for the NS1 D2N^†^ and R227K^†^ mutants that also possess NEP mutations. These results indicate that the majority of NS1 mutations (10 of 12; except mutants V180A^†^ and V226I) selected upon HK-wt virus passage in the mouse were adaptive *in vitro*, and increased viral replicative fitness by up to 100 fold in mouse cells (Fig. 6, left panel).

### Mouse-adapted NS1 mutations increase viral growth in IFN-β primed cells *in vitro*

Given that the NS1 protein is an interferon antagonist, we next asked whether NS1 mutations selected upon mouse adaptation affected resistance to interferon beta (IFN-β) pre-treatment upon *in vitro* infection. We assayed viral growth in M1 cells as per the previous protocol, however prior to infection the cells were primed with 1000 U/mL recombinant mouse IFN-β for 24 h to induce an antiviral state. rHK-wt viral yield was reduced by 16 fold by IFN-β pre-treatment at 72 hpi, where viral yield reached a maximum of 2.3×10^2^ PFU/mL in IFN-β primed cells relative to 3.8×10^3^ PFU/mL yield in untreated cells (Fig. 6). NS1 mutants D2N^†^, L98S, M106I + L98S, M106V + M124I, and D125G all significantly enhanced viral growth throughout the course of infection in IFN-β primed cells when compared to rHK-wt (p<0.001, p<0.01, p<0.01, p<0.05, p<0.001, respectively; two-tailed paired t-test) (Fig. 6, right panel). Viral replication in IFN-β primed cells was enhanced at early times in infection (12–24 hpi) for NS1 mutants V23A, F103L, M106I, M106V, and R227K^†^ compared to rHK-wt (p<0.01, by two-tailed, paired t-test). The three NS1 mutants that produced the greatest viral yield in IFN-β primed cells, M106I + L98S, M106V + M124I, and D125G, exceeded rHK-wt maximum yield obtained at 72 hpi by 100, 78, and 12 fold, respectively. These data indicate that the majority of NS1 mutants selected upon mouse adaptation (10 of 12; all except mutants V180A^†^ and V226I) enhanced viral growth in both naive and IFN-β primed mouse cells *in vitro*, consistent with a role for IFN antagonism in enhanced growth *in vitro*.

### Mouse-adapted NS1 mutations enhance HK virus mRNA transcription *in vitro*

**Figure 7:**
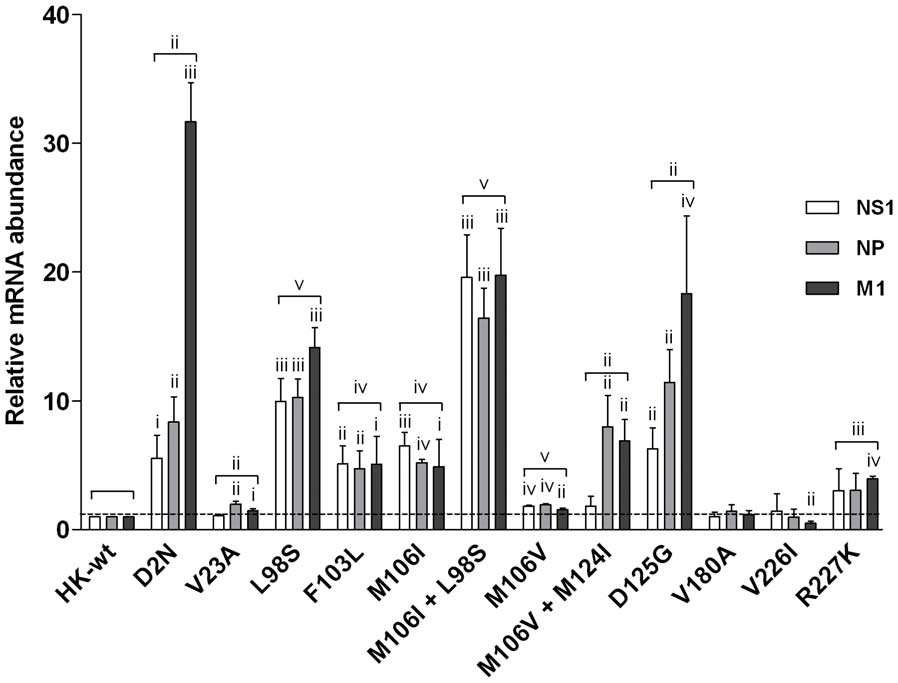
Mouse-adapted NS1 mutations enhance viral mRNA production in mouse cells *in vitro*. Mouse M1 cells were infected in triplicate at an MOI of 2 with rHK NS mutants or rHK-wt virus, and total RNA isolated at 8 hpi was reverse transcribed using primers specific for viral mRNA. Real-time PCR (qPCR) was performed to quantify NP, M1 and NS1 viral mRNA levels. Results were normalized by β-actin levels, and presented as values relative to rHK-wt mRNA levels. Data represent the means ± SD (two-tailed student's paired t-test) for NP, NS1 and M1 mRNA relative levels (indicated by bracket) or two-tailed student's t-test for individual mRNA relative values (i p<0.05; ii p<0.01; iii p<0.001; iv p<0.0001; v p<0.00001).

Since the majority of the NS1 mutants increased replication of the HK virus in mouse cells *in vitro*, we next determined whether this enhanced replicative fitness was due to increased viral mRNA production. Confluent monolayers of M1 cells were infected in triplicate with rHK-wt or rHK NS mutants (MOI = 2) and total RNA was extracted from the cells at 8 hpi for real-time quantitative RT-PCR analysis of NS1, NP (a representative early viral transcript), and M1 (a representative late viral transcript). Figure 7 shows the average production of mRNA normalized to the HK-wt yield ± standard deviation for each of the NS1, NP, and M1 genes. Upon statistical analysis pairing the groups of 3 genes, the relative mRNA abundance of the three viral transcripts tested was significantly higher than rHK-wt for all NS1 mutants (p<0.01, two-tailed paired t-test) with the exception of mutants V180A^†^ and V226I (Fig. 7). The greatest increases in mRNA accumulation were seen for the D2N^†^, M106I + L98S, and D125G mutants (>10 fold). All assayed viral mRNAs were shown to be significantly upregulated for NS1 mutants D2N^†^, L98S, F103L, M106I, M106I + L98S, M106V, and D125G (Fig. 7, p<0.05- p<0.000001, student's t-test). Several NS1 mutants also affected the ratio of NS1, NP and M1 mRNA transcript abundance. The ratio of M1 mRNA was significantly greater than NS1 mRNA for NS1 mutant L98S (p<0.05), and levels of M1 and NP mRNA were both significantly higher than NS1 mRNA for NS1 mutants M106V + M124I and D125G (p<0.05, NP>NS1 mRNA; p<0.01, M1>NS1 mRNA) (Fig. 7).

**Figure 8:**
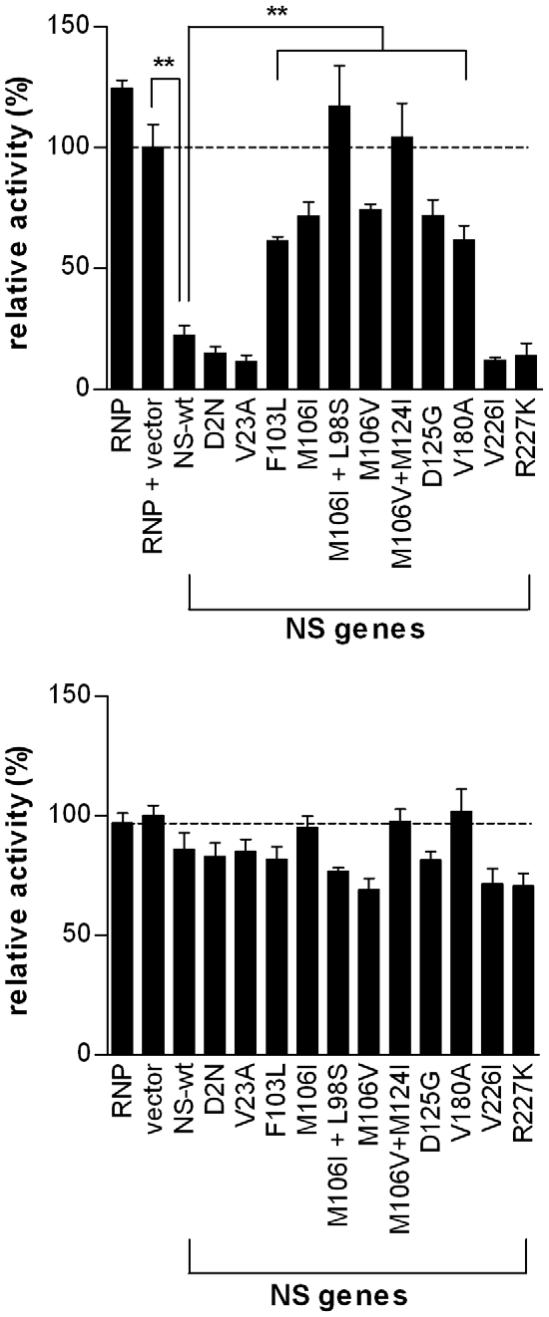
RNA polymerase mini-genome assay. Viral RNA polymerase activity was measured in HEK 293-T cells expressing mutant or HK-wt NS genes as well as HK-wt PB2, PB1, PA, NP, and firefly luciferase driven by the NP promoter. (A) Relative polymerase activity was calculated as the ratio of firefly luciferase to renilla luciferase (under SV40 promoter control) luminescence and presented as means ± SD for three experiments (**p<0.01, student's t test). (B) NS mutants do not affect host gene expression. Renilla luciferace activity in cells transfected with HK RNP is not affected by NS mutant expression (p>0.05, student's t-test) compared to HK wt NS expression.

To further support the findings of NS1-mediated increased RNA synthesis, we next performed a luciferase mini-genome assay to determine the effect of mutant NS gene segments on viral RNA polymerase activity. RNA polymerase activity was measured in HEK 293-T cells by detecting luciferase activity expressed from an influenza firefly luciferase mini-genome plasmid expressed via human RNA polymerase I transcription of luciferase flanked by the NP gene non-coding regions [5] in the presence of HK-wt PB2, PB1, PA, NP and various HK-wt or mutant NS genome segments, as detailed in methods. Virus-dependent transcription of firefly luciferase was standardized to the level of renilla luciferase expressed via the constitutive SV40 promoter of the PRL-SV40 plasmid. When comparing polymerase activity in the presence of HK-wt NS and NS mutants, NS1 mutants F103L, M106I, M106V, D125G, and V180A^†^ all increased polymerase activity by >2.5 fold (p<0.01, student's t test) (Fig. 8). The NS1 mutants with the highest increases in viral polymerase activity, M106V+M124I and M106I+L98S, both increased activity >4.5 fold compared to rHK-wt NS (p<0.01, student's t-test) (Fig. 8), which indicated enhanced activity due to the second mutation in both instances. Renilla expression via the host RNA polymerase II was not significantly different from HK-wt for any of the NS gene mutants (Fig. 8). Thus all NS1 mutants that demonstrated increased viral polymerase activity in the RNA polymerase minigenome assay also demonstrated increased viral mRNA production in infected cells (Fig. 7) with the exception of NS1 mutant V180A^†^. The increased transcription seen in infected M1 cells for the D2N^†^, V23A, and R227K^†^ was not associated with increased RNA polymerase activity and suggests an indirect mechanism of action on RNA synthesis. The V226I mutant did not demonstrate increased RNA synthesis by either assay indicating that the increased replication *in vivo* may be independent of enhanced RNA synthesis.

Expression of the HK-wt NS gene segment was also shown to decrease viral polymerase activity when compared to the empty vector control (p<0.01) (Fig. 8), which suggested a negative regulatory effect due to the NS1 and/or NS2/NEP genes expressed from the NS construct. By using pLLB vector plasmids that express the NS gene segment, versus NS1 or NS2/NEP genes alone (see methods), we found that expression of the NS gene segment inhibited viral polymerase activity due to NS1 and NS2/NEP gene expression (Fig. S2). Moreover, expression of NS1 alone was shown to significantly reduce renilla luciferase activity, however upon co-expression with NEP in the NS gene segment, renilla luciferase activity was similar to the vector control (Fig. S2). Further analysis is required to determine the mechanism of polymerase activity regulation by the NS1 and NS2/NEP proteins.

This data shows NS1 mutants selected upon mouse adaptation increase viral gene expression at the level of transcription both in the luciferase mini-genome assay and in infected mouse cells. In addition some NS1 mutants also differentially affect transcriptional regulation.

### Mouse-adapted NS1 mutations enhance HK virus protein synthesis *in vitro*, in untreated and IFN-β primed M1 cells

Given that the NS1 protein enhances viral protein synthesis as well as antagonizes IFN-mediated inhibition of virus replication, we hypothesized that the increase in viral growth of the NS1 mutants in IFN-β primed mouse cells compared to rHK-wt virus may be due to increased IFN-resistant viral protein synthesis. To test whether the MA NS1 mutations conferred increased viral protein synthesis, and whether these mutations influenced resistance of viral protein synthesis to the IFN-β-mediated anti-viral response, we evaluated the level of M1 and NS1 viral protein synthesis by pulse labelling with ^35^S-labeled cysteine and methionine residues during the course of infection in M1 cells (MOI = 2), both in untreated and in IFN-β primed cells that were treated with 200 U/mL mouse IFN-β for 24 hours prior to infection.

**Figure 9:**
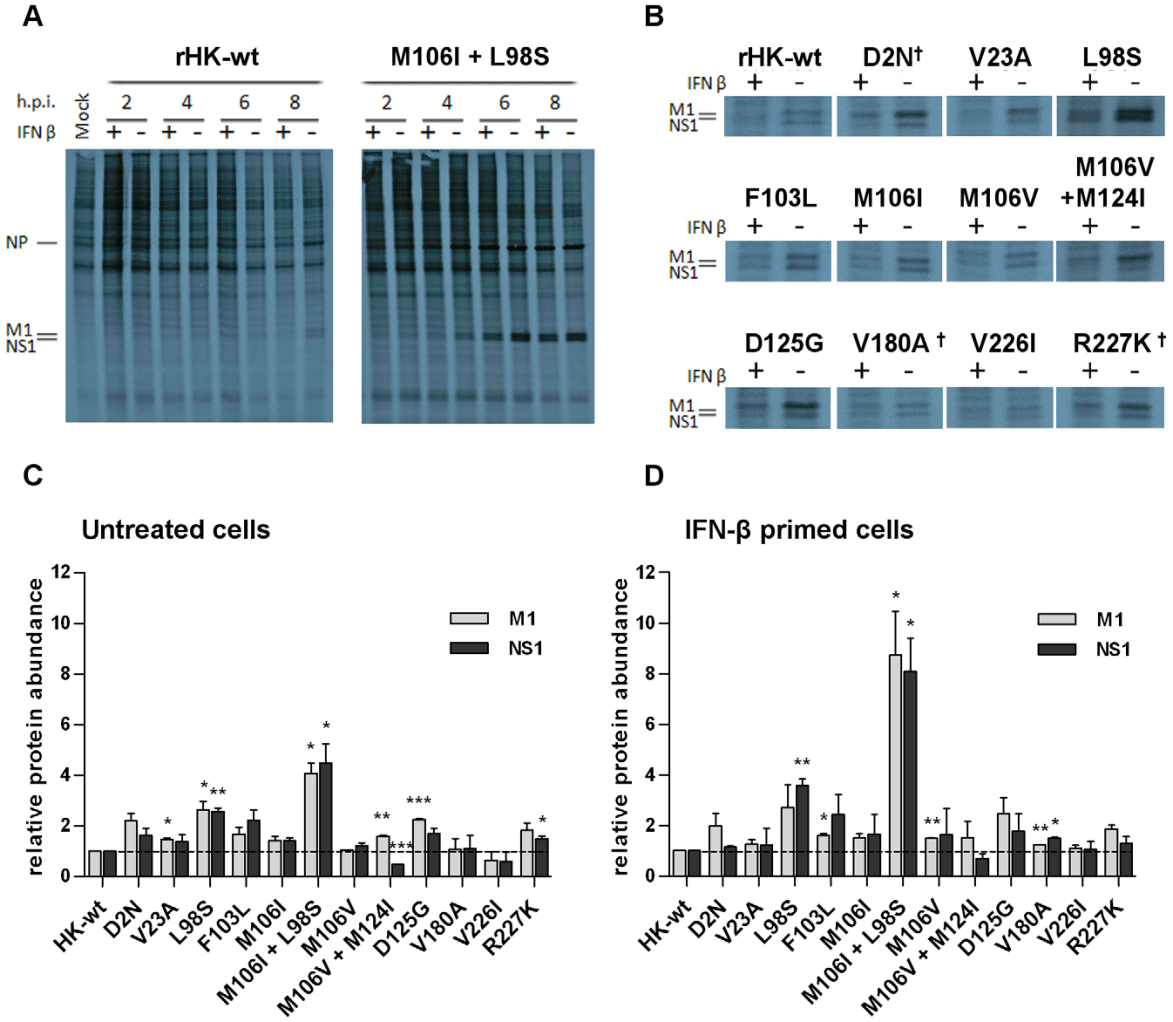
Mouse-adapted NS1 mutations enhance virus protein synthesis in untreated and IFN-β primed mouse cells *in vitro*. M1 cells were left untreated or were pre-treated with 200 U/mL murine IFN-β for 24 hours, then infected with rHK NS MA or HK-wt virus at an MOI of 2. At 2, 4, 6, and 8 hpi the cells were pulsed with ^35^S for one hour then lysate was collected in SDS buffer. Autoradiography of collected samples are shown for (A) full time courses of HK-wt and rHK NS M106I + L98S and (B) 8 hour time points of all NS mutants. (+) and (−) symbols indicate whether cells were pre-treated with IFN-β. NP, M1 and NS1 protein positions are indicated, and were verified by western blot (data not shown). Data shown are representative of two independent experiments, which were each subject to densitometry analysis. Data represent the means ± SE (*p<0.05, **p<0.01, *** p<0.001; two-tailed student's t-test) for NS1 and M1 protein band density relative to rHK-wt in untreated cells (C) and in IFN-β primed cells (D).

Although rHK-wt produced detectable M1 and NS1 proteins in untreated cells beginning at 8 hpi, and at a reduced level in IFN-β primed cells, viral protein bands were prominent for the double mutant M106I + L98S starting at 4 hpi in both untreated and IFN-β primed cells (Fig. 9a). Similar to the double mutant, the NS1 mutant L98S produced detectable viral proteins starting at 4 hpi (data not shown). Given that the remaining NS1 mutants did not demonstrate increased viral protein synthesis relative to rHK-wt before 8 hpi (data not shown), quantification of M1 and NS1 protein bands was performed for 8 hpi samples from both cell conditions as mean values ± standard error for duplicate experiments (Fig. 9a–d). With respect to HK-wt and NS1 mutant infection, IFN-β pre-treatment was shown to suppress viral protein synthesis compared to infection in untreated cells (Fig. 9a–b). Although there was a trend to increased synthesis for one or both of the NS1 and M1 proteins for all the NS1 mutants except mutants V180A^†^ and V226I relative to rHK-wt at 8phi in untreated cells, significantly increased levels of viral protein were seen for NS1 mutants V23A (M1 protein, p<0.05), L98S (M1 protein, p<0.05; NS1 protein, p<0.01), M106I+L98S (M1 and NS1 proteins, p<0.05), M106V+M124I (M1 protein, p<0.01), D125G (M1 protein, p<0.001), and R227K^†^ (NS1 protein, p<0.05) (Fig. 9a–c). NS1 mutants L98S and M106I+L98S both produced the most viral protein; compared to rHK-wt, L98S produced >2.5 fold more M1 and NS1 protein, and mutant M106I+L98S produced >4 fold more M1 and NS1 protein (Fig. 9c).

In IFN-β primed cells, both NS1 mutants L98S and M106I + L98S produced the highest levels of viral protein compared to rHK-wt; NS1 protein levels were >2.5 fold higher for NS1 mutant L98S (p<0.01), and both M1 and NS1 levels were >8 fold higher than rHK-wt for mutant M106I + L98S (p<0.05) (Fig. 9d). The remaining mutants, with the exception of NS1 mutants V23A and V226I, demonstrated a trend of increased viral protein synthesis that was significantly greater than rHK-wt for NS1 mutants F103L (M1 protein, p<0.05), M106V (M1 protein, p<0.01) and V180A (M1 protein, p<0.01; NS1 protein, p<0.05) (Fig. 9d).

**Figure 10:**
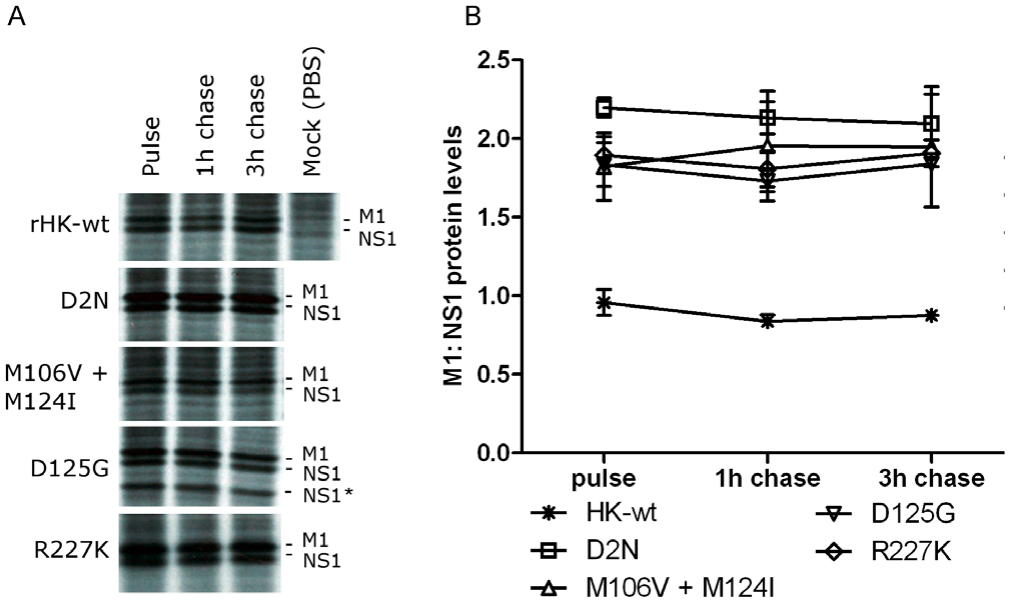
NS1 mutants with altered M1∶ NS1 protein production ratio do not demonstrate altered viral protein half lives. M1 cells were infected with specified rHK NS1 mutants or HK-wt virus at an MOI of 2. At 8 hpi the cells were pulsed with ^35^S for one hour. For pulse samples, the lysate was then collected in SDS buffer. For chase samples, the pulse medium was replaced with serum-free MEM then cell lysate was collected 1 or 3 hours later (n = 3). (A) Autoradiography of collected samples, indicating bands corresponding to M1 or NS1 protein, as verified by Western blot, (B) protein bands were quantified by PhosphorScreen signal amplification, then band intensity quantification using GE Imagequant software. Data represent the means ± SD of the ratio of M1 to NS1 protein levels. Statistical analysis was performed, however no virus induced a statistically significant (p<0.05, student's t test) difference in the ratio of M1∶NS1 from pulse to chase.

The ratio of M1 protein production to NS1 protein production was significantly affected for NS1 mutant M106V+M124I. Whereas rHK-wt produced equal band intensities for M1 and NS1 proteins at 8 hpi in untreated cells, NS1 mutant M106V + M124I produced significantly more M1 protein than NS1 protein (p<0.01) (Fig. 9b–c). NS1 mutants D2N^†^, D125G, and R227K^†^ also exhibited a trend of increased M1 protein relative to NS1 protein although the difference was not statistically significant (Fig. 9b–c). To determine whether the observed differences in M1 to NS1 protein ratio was due to decreased NS1 protein stability, we performed a radioactive pulse-chase experiment. Briefly, M1 cells were infected with rHK-wt, and NS1 mutants M106V + M124I, D2N^†^, D125G, and R227K^†^ as per previous experiment. At 8 hpi the cells were pulsed with ^35^S-labeled cysteine and methionine, then at 9 hpi, the cells were either collected (pulse samples) or were chased with complete MEM for 1 or 3 hours (n = 3) (Fig. 10a). The data demonstrate that compared to rHK-wt NS1 and M1 protein stability, the NS1 mutants tested did not affect the stability for these proteins, measured as the ratio of M1 to NS1 protein levels in each condition (Fig. 10b). An additional protein band of 20 kDa was detected with NS1 antibody in D125G infected cells and is the subject of further investigation (Selman, Forbes, Dankar, and Brown, in preparation). Taken together, these observations indicated that several NS1 mutations altered the temporal regulation of viral protein production in a gene-dependent manner (Fig. 9b–c, Fig. 10). The observation that the relative increases in RNA synthesis were greater than the relative increases in protein synthesis suggests that restrictions in protein synthesis are limiting gene expression in the mouse system.

The increased viral mRNA levels, viral protein synthesis and IFN-β resistance of viral protein synthesis were consistent with the enhanced rates and levels of viral replication in mouse M1 cells and also demonstrated an increased resistance to type I IFN antiviral mechanisms (Fig. 6, 7, 8, 9).

## Discussion

Although the NS1 protein has multiple binding partners and functions that promote viral replication, including antagonism of the IFN response, it is not clear which functions are altered or enhanced in virulent variants. We employed mouse adaptation as an unbiased approach to identify adaptive mutations in the NS1 gene, an approach that has identified adaptive mutations in the polymerase and receptor binding protein that have been confirmed to increase replication and virulence [5], [7], [45], [46]. Consistent with the multiple roles of the IAV NS1 protein in supporting virus replication, we demonstrated that the selection of A/Hong Kong/1/1968 virulent variants in the mouse led to identification of key NS1 mutations responsible for adaptation and virulence in a novel host. By generating recombinant HK viruses differing in their NS gene segment we demonstrated that individual and multiple MA NS1 mutations increased viral replication, viral gene expression, virulence in the mouse, and antagonism to IFN-β, both by limiting the amount of the antiviral cytokine produced, and by limiting the effect of IFN-β action on virus replication (summarized in Table 1).

**Table 1:** Adaptive properties of rHK viruses expressing mouse-adapted NS mutations^5^.

	HK-wt	D2N^†^	V23A	L98S	F103L	M106I	M106I + L98S	M106V	M106V + M124I	D125G	V180A^†^	V226I	R227K^†^
**% mortality**	0	83	0	20	0	20	40	20	0	20	20	0	0
**weight loss** 1	1	3.4	1.9	1.9	2.1	3.8	3.3	3.1	2.7	2.9	2.0	1.0	1.7
**Lung IFN-β** 2	1	1.4	0	0	0.12	0.54	1.0	0	0	0.03	0	0	0
**Lung titre 1 dpi**	1	12.0	16.4	1.0	1.0	1.3	7.6	1.1	0.02	13.4	0.92	0.74	2.35
**Lung titre 3 dpi**	1	0.75	0.38	1.5	0.57	1.1	2.3	3.2	0.03	4.1	2.2	5.3	0.79
**CPSF binding**	1	0.87	2.1	0.08	0.02	0	0	0	0	0	0	1.1	2.2
**Yield ** ***in vitro*** 3	1	12.5	3.9	10.7	2.8	1.6	121	1.3	53.9	20.5	1.0	0.74	1.2
**Yield +IFN** 2	1	8.1	6.9	3.4	1.6	1.4	100	1.2	78.4	12.0	0.50	0.23	0.90
**viral mRNA** 4	1	13	1.5	11.4	5.0	5.5	18.6	1.8	5.6	13.4	1.2	1.0	3.4
**RNA polymerase**	1	0.7	0.5	nd	2.7	3.2	5.2	3.3	4.7	3.2	2.8	0.5	0.6
**viral protein** 3	1	1.9	1.4	2.6	2.0	1.4	4.3	1.1	1.0	2.0	1.1	0.6	1.7
**viral protein +IFN** 3	1	1.6	1.2	3.1	2.0	1.6	8.4	1.6	1.1	2.1	1.4	1.1	1.6

1 Assessed at respective dpi with maximum weight loss for each respective virus.

2 Fold change values of lung IFN-β levels 1 day pi.

3 Relative maximum titre obtained 72 hpi (Fig. 6).

4 Fold change of overall mRNA expression (NP + M1 + NS1; Fig. 7) or overall viral protein expression (M1 + NS1; Fig. 9c–d), respectively.

5 All fold values are relative to rHK-wt phenotype; where 0 represents below the limit of detection by the respective assay, with the excetion of % mortality, which is expressed as values independent to rHK-wt. (nd, not determined).

**Figure 11:**
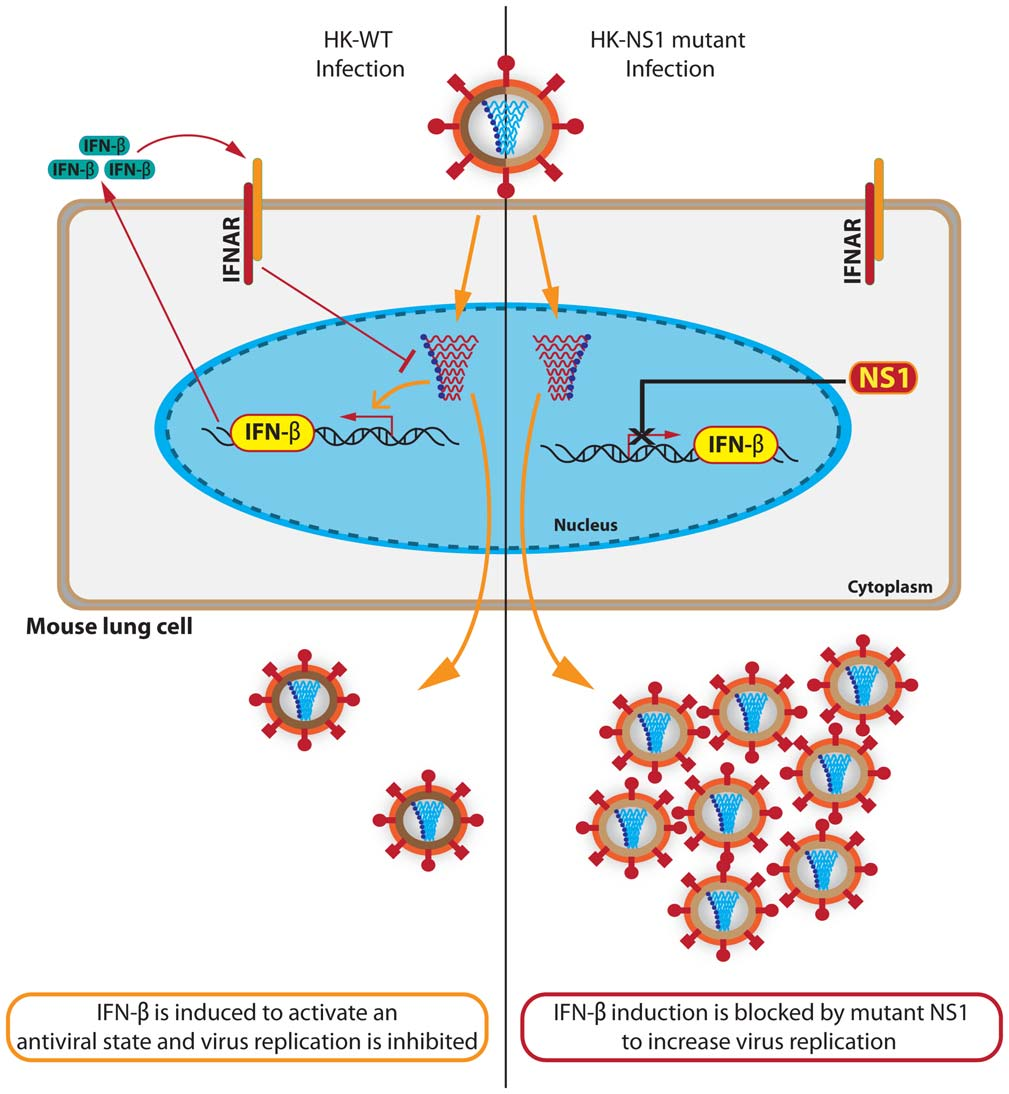
Model of adaptation of human HK-wt influenza virus to the mouse lung to enhance IFN-β antagonism. Mouse adaptation involves the selection of NS1 mutants with enhanced IFN-β antagonism, so that upon infection of mouse lung cells *in vivo* the mutant NS1 gene reduces IFN-β gene (in yellow) activation in the nucleus and thus IFN-β protein (green) production and release. Therefore adaptive mutations prevent the IFN-β induction of an antiviral state that is mediated through binding of to the IFN-α/β receptor (IFNAR). This model proposes that reduced IFN-β production leads to a reduction in the antiviral state and greater virus yield.

Whereas very few NS1 mutations have been identified in genomic analyses of other MA variants, we have employed high dosages and increased the number of passages, which resulted in virulent variants with mutations in the NS1 gene [5]. Here we show that all mutant NS1 genes increased viral replication in mouse cells and/or mouse lungs indicating that they have acquired adaptive properties. Increased replicative fitness in untreated mouse cells was seen at the level or rate of virus growth as well as virus gene expression (at both the mRNA and/or protein levels), for all NS1 mutants except V180A^†^ and V226I. (Fig. 6, 7, 8, 9, Table 1). In IFN-β primed cells, all NS1 mutants with the exception of V226I exhibited enhanced yield and/or replication kinetics (Fig. 6, Table 1). Moreover, viral protein synthesis in cells primed with IFN-β was enhanced for 5 of 12 NS1 mutants (L98S, F103L, M106I+L98S, M106V, and V180A^†^) (Fig. 9d). Analysis of the role of mouse-adapted NS1 mutations in the mouse lung *in vivo* (summarized in Table 1) demonstrated increased virulence and disease severity for most mutations, where all NS1 mutants with the exception of V226I and R227K^†^ significantly increased body weight loss compared to rHK-wt from days 1–6 pi (Fig. 2b, Table 1). Moreover, the amount of IFN-β produced in the mouse lung was significantly less than rHK-wt for most NS1 mutants, and of these mutants, the majority induced greater viral yield relative to rHK-wt levels (Fig. 4, Table 1), which suggests a model for selection of NS1 mutations upon host adaptation, where mutations that enhance IFN-β antagonism demonstrate increased viral yield (Fig. 11). Lower IFN-β induction in the mouse lung upon intranasal infection was consistent with the observation of a prototype mouse-adapted strain; A/PR/8/34 (H1N1), that induced low levels of IFN-β in the lung (21.7±8.7 pg/g) relative to the A/Hong Kong/1/1968 (H3N2) human clinical isolate that induced 668.5±353.5 pg/g upon intranasal inoculation of 1×10^5^ PFU dose in BALB/c mice (data not shown). However, in the case of the two NS1 mutants that induced the greatest mortality, D2N^†^ and M106I + L98S, viral yield in the lung was 12 and 8 fold greater than rHK-wt (1 dpi), while the corresponding level of IFN-β concentration was significantly increased but only by 50% (p<0.01) or was similar to rHK-wt, respectively (Fig. 4, Table 1). Both D2N^†^ and M106I + L98S NS1 mutants induced greater pathology in the lung and virus spread in the lung compared to rHK-wt (Fig. 3). Taken together, these findings identify multiple adaptive paths employed by NS1 mutations selected upon human IAV passage in the mouse. We speculate that NS1 mutations that confer decreased IFN-β induction in the mouse lung may in some cases allow replication to higher titres before inhibitory levels of IFN are reached, ultimately resulting in greater spread and infection in the lung and correspondingly, increased disease severity and mortality (as seen for the majority of mutant NS1 genes) (Fig. 2, 3, Table 1). To our knowledge, this is the first demonstration of adaptive mutations in the NS1 gene that result in greater antagonism of IFN induction, and one of the few reports that describe increased antagonism of the IFN-induced antiviral response and virulence [9]. Adaptive mutations in the NS1 gene that lead to decreased IFN production could block IFN induction at the level of RIG-1-mediated viral RNA sensing where NS1 amino acids at residues 96 and 97 have been associated with this mechanism to interfere with IFN induction [14], especially for adaptive NS1 mutations proximal to this region (Fig. 5a). Alternatively, there is a possibility that reduced IFN-mediated signaling and activation of IFN-inducible effector genes (including the autocrine amplification of type I IFN production) could also be affected by adaptive NS1 mutations [15]. Future research on the effect of NS1 mutants on these antiviral signaling pathways will identify the mechanism(s) employed to enhance IFN antagonism.

### NS1 suppression of IFN-β induction is independent of ability to bind CPSF30-F2F3

A primary function of NS1 protein is to inhibit activation of IFN mRNA transcription by interfering with RIG-I-mediated detection of viral RNA. However, numerous studies have shown that the ability of NS1 to inhibit general host gene expression is dependent upon the ability to bind the F2F3 domain of the post-transcriptional processing factor CPSF30, and that this interaction leads to the suppression of host mRNA processing, including type I IFN mRNA [17], [18], [34]. Some studies have shown that the loss of CPSF binding through *in vitro* mutagenesis is associated with increased IFN induction and attenuated growth for A/Udorn/1/1972 (H3N2) [18] or its reassortant possessing the 1997 H5N1 NS1 gene [34]. However, other studies of natural variants that do not bind CPSF show that these viruses (mouse-adapted A/PR/8/34 (H1N1) [47] and pandemic 2009 H1N1 isolates [48]) maintain the ability to block IFN-β production but lose the ability to block general host gene expression; where restoration of CPSF30 binding (that required the mutagenesis of 2 or 3 aa, respectively) resulted in slightly increased or reduced virulence, respectively. Consistent with these data we observed that NS1 mutations selected on mouse adaptation in many cases reduced or blocked CPSF30-F2F3 binding but increased replication, RNA polymerase activity, virulence and IFN antagonism. Specifically, NS1 mutations at sites L98S and F103L significantly decreased binding to CPSF30-F2F3 and mutations at aa site 106 (M106I, M106I + L98S, M106V, M106V+M124I) as well as NS1 mutants D125G and V180A^†^ abolished binding to CPSF30-F2F3 (Fig. 5c–d). Moreover, all NS1 mutants with suppressed CPSF30-F2F3 binding with the exception of mutant M106I + L98S were able to reduce the amount of IFN-β produced *in vivo* 1 dpi to levels ≤ rHK-wt, while attaining viral yields that were ≥ rHK-wt in the mouse lung demonstrating a lack of correlation between CPSF-binding and IFN-β synthesis (Fig. 4, Fig. 5). Our data are consistent with the recent study of Steidle *et al.* (2010), where a mutant A/PR/8/34 (H1N1) virus with restored binding to CPSF30 due to S103F+I106M substitutions was rendered deficient in binding CPSF30 upon further amino acid substitution (G184R) but still remained capable of inhibiting IFN production [47]. Because host mRNA transcripts are required as primers for IAV transcription [49] and many host genes are required for viral replication [50], increased general host gene expression by reduced NS1:CPSF30 binding may be adaptive in a new host environment where virus replication is deficient. In this regard we have observed a reciprocal relationship between CPSF binding and RNA polymerase activity where viruses that have lost CPSF binding demonstrated increased RNA polymerase activity (Fig. 5 and 8, Table 1). Further research on the effect of MA NS1 mutants on these properties is expected to identify alternative mechanism of IFN antagonism.

### MA NS1 mutations enhance viral gene expression

All NS1 mutants except V180A^†^ and V226I were found to increase viral mRNA synthesis in infected cells, and 7 of 11 MA NS gene segments were found to increase viral polymerase activity in HEK 293-T cells, which in several cases correlated with increased viral protein synthesis compared to rHK-wt (Fig. 7, 8, 9, Table 1). This indicates that NS1 mutations selected upon mouse adaptation increase viral gene expression indicating a functional interaction between the NS1 gene and polymerase activity due to direct or indirect interactions with viral polymerase components. This hypothesis is supported by numerous studies that have linked NS1 action with the ability of influenza virus to synthesis viral RNA or associate with components of the polymerase complex [23], [29]–[34]. Increased viral protein synthesis was also shown for HK NS1 F103L and M106I mutations when expressed on the A/PR/8/34 (H1N1) backbone [7] and given the location of these and D2N^†^, V23A, L98S and M106V MA NS1 mutations within binding domains for cellular factors involved in translation (eIF4GI, PABPI; Fig. 5a), we cannot rule out the possibility that enhanced interaction with these factors may also contribute to increased levels of viral protein synthesis.

In addition to increased yields of viral mRNA and protein, we observed differential regulation of M1 and NS1 protein synthesis relative to rHK-wt levels. NS1 mutants D2N^†^ and D125G induced lower levels of NS1 protein relative to M1 protein, that was also demonstrated at the level of mRNA synthesis. Pulse chase protein labeling demonstrated this phenotype was not due to alteration of NS1 protein stability (Fig. 10). Loss of temporal regulation of transcription has been shown for alanine mutations at position 123+124 in NS1 [23]. Further research on the effect of MA NS1 mutants that increase viral protein synthesis (Fig. 9) on binding to cellular translation factors is required to determine the mechanism of viral protein synthesis enhancement.

### MA NS mutations which induce mutations in the nuclear export protein

Several NS1 mutations reside in the overlapping reading frame for the nuclear export protein (NEP), also known as non-structural protein 2 (NS2), a spliced gene product of the mRNA expressed from gene segment 8. The NEP is known to function in nuclear export of viral ribonucleoproteins (vRNPs) in association with the matrix protein (M1) [51]–[54]. The NEP protein has also been linked to regulation of viral transcription and replication of the viral genome in a mechanism that is independent of vRNP nuclear export [55]. Of the MA viruses with NS1 mutations, 4 contained NEP mutations (Fig. 1a). The mouse-adapted variants MA62 and MA63 possessed the NS1 mutation V180A, which concurrently induced the NEP mutation S23P. The NS gene segment of MA93 (NS1 D2N) that induced the highest level of virulence when expressed as a rHK NS mutant (Fig. 2), contained two NEP mutations, D2N and E108K. Since both the NS1 and NEP proteins have the first 10 amino-terminal aa in common, it is possible that NS1 and NEP mutations within this region involve a common mechanism of action. MA112 (R227K) also contained NEP mutation G70S due to the same nucleotide substitution in the overlapping NEP gene. Currently we are unable to separate the effects of these NEP mutations from their associated NS1 mutations when expressed as a recombinant NS mutant virus due to the restrictions of independently mutating overlapping codons. Future studies could employ a modified gene 8 construction that separates the NS1 and NEP genes as in Perez *et al.* (2010), where the two NS1 and NEP genes could be independently engineered to unlink the incorporation of NS1 and NEP mutations [56].

### Mouse adapted NS1 mutations: Pattern of Selection

Adaptation of the human isolate A/Hong Kong/1/1968 to the mouse resulted in strong selection for highly virulent mouse-adapted variants, 18 of 39 which contained mutations in the NS1 gene [5]. Two incidences of convergence occurred between separately evolved virus populations, where two independently passaged virus populations both selected mutations at amino acid location 106 (M106V, and M106I, respectively), and another two independently passaged populations selected the D125G mutation [5] which was also independently selected in the H3N2 mouse adapted variant of A/Aichi/2/68 [44]. The adaptation of duck H9N2 virus to quail has also been shown to select a mutation at position 106 (M106T) [57] that further supports that this site is an important modulator of NS1 gene function. We also obtained mutations in adjacent amino acid positions, 124+125 and 226+227, suggesting that these are focal adaptive regions within the NS1 gene. A pattern of sequential NS1 mutation was seen where 3 of 3 variants in one passaged virus population possessed mutation M106I and 2 of 3 possessed a second mutation, L98S [5]. Both NS1 mutants M106I and L98S increased viral fitness and virulence compared to rHK-wt and together demonstrated synergy where the phenotype of the double mutant M106I+L98S was significantly increased (Fig. 2, 3, 4, 6, 7, 8 and 9). This suggests that double mutations in NS1 may have the greatest phenotype by compensating or synergizing properties that enhance replication in the new host. In another passaged population, the presence of the NS1 mutation M106V in all 3 clones with the additional M124I mutation in clone 3 [5] was associated with increased *in vitro* phenotypes of the M106V+M124I mutant relative to the M106V mutant, and was consistent with a sequential selection of synergistic mutations.

Sequence comparisons with NS1 genes derived from highly virulent IAV isolates identified numerous incidences of convergence between such strains and our MA strains. A/Hong Kong/156/1997(H5N1) is convergent with the MA NS1 mutations at amino acid sites 23A, 103L, and 106I. The F103L and M106I mutations have been demonstrated to confer increased virulence in the A/Hong Kong/156/1997 NS1 gene when expressed on the A/Puerto Rico/8/1934 (H1N1) and the A/Chicken/Beijing/1/1995 (H9N2) NS1 gene expressed on the A/WSN/33 (H1N1) mouse-adapted genetic backbone as recently shown by Dankar *et al.* (2011) [7]. As mentioned earlier we have shown that the HK NS1 V23A mutation enhanced virulence 6 fold [46]. The NS1 mutation D125G, which resides within the NS1 PKR binding domain, was also selected upon adaptation of the human influenza virus isolate A/Aichi/2/68(H3N2) to high virulence in the BALB/c mouse [44], which suggests that NS1 interactions with PKR may confer an adaptive advantage upon evolution in a new host. NS1 mutations 226I and 227K are also shared with the 1918 Spanish influenza A/Brevig Mission/1/1918 (H1N1) NS1 gene [58], which has been shown to contribute to virulence in infected Macaques [59] and delay IFN induction in ferrets [60]. Although the R227K and V226I mutations were shown to be adaptive *in vivo* when expressed as single mutations (Fig. 4), neither induced significant increases of morbidity in the mouse (Fig. 2), however it is possible that these mutations contribute to the virulence of the 1918 pandemic in the context of the 1918 NS1 protein or other 1918 viral genes.

In summary, all mutant NS1 genes selected upon IAV passage in the mouse were adaptive and increased replication that was in most cases associated with increased virulence in the mouse. Increased replication was associated with increased gene expression and IFN antagonism. Consistent with the role of NS1 in IAV as a virulence factor we have identified NS1 mutations that control adaptive properties associated with host adaptation and virulence.

## Materials and Methods

### Ethics Statement

All *in vivo* research was performed in accordance with the guidelines of the Canadian Council on Animal Care (CCAC) as outlined in Care and Use of Experimental Animals, Vol. 1., 2nd Ed (1993), which are internationally recognized as “best practices” by the International Council for Laboratory Animal Science (ICLAS). The animal study protocol was approved by the University of Ottawa Animal Care Committee (Protocol No. BMI-85). All efforts were made to minimize suffering, and mice were humanely euthanized upon experimental endpoint (when infection resulted in greater than 30% body weight loss accompanied by respiratory distress).

### Cells and viruses

MDCK (Madin-Darby canine kidney, Health Canada, Ottawa, Canada) and M1 (Mouse kidney epithelium, ATCC) cells were maintained in minimum essential medium (MEM), and 293T (Human embryonic kidney, ATCC) cells were maintained in Dulbecco's minimal essential medium (DMEM) (Gibco, Invitrogen), both supplemented with L-glutamine (2 mM), Penicillin (100 U/ml), Streptomycin (100 ug/ml) and fetal bovine serum (FBS) (10%). All cells were incubated at 37°C in the presence of 5% CO_2_. The H3N2 human influenza isolate A/Hong Kong/1/1968 (HK-wt) was obtained from the Laboratory Center for Disease Control (Health Canada, Ottawa, Canada). Viruses were plaque purified in MDCK cells and grown in the allantoic cavity of 10-day-old SPF chicken embryos (Canadian Food Inspection Agency, Ottawa).

### Directed evolution of influenza A virus in the mouse lung

The generation of mouse-adapted (MA) variants after 20 or 21 passages (MA20 or MA21) in the CD-1 mouse lung was described previously [42], [5]. All MA21 viruses were designated as MA (mouse passage population #)-(clone #) and referred to by their indicated abbreviation (Fig. 1a).

### Plasmid Construction

Genome segment 8 cDNA of A/Hong Kong/1/1968 was inserted into the bi-directional pLLB vector by ligation-independent cloning [61]. Each MA NS mutation was introduced into the HK NS gene (Genbank accession no. CY033005) by PCR-based site-directed mutagenesis. All of the plasmid constructs were sequenced to ensure the absence of unwanted mutations. The F2F3-FLAG pET17b plasmid under T7 promoter control was constructed using homologous recombination. Briefly, pCAGGS-CPSF30-F2F3-FLAG (obtained gratefully from L. Martinez-Sobrido, Mt. Sinai School of Medicine) was cloned using primers specific to CPSF-F2F3-FLAG with homologous ends to pET17b sequence upstream of the T7 promoter. The F2F3-FLAG construct was subject to homologous recombination with pET17b using DH5α E.coli competent cells. Positive colonies were subject to sequencing to verify proper insertion of construct. The CPSF30 F2F3 gene was of human origin, however the F2F3 portion of CPSF30 is completely conserved in the mouse [5].

### Virus Rescue

Each MA-NS pLLB plasmid was incorporated into the parental HK-wt genomic backbone (Genbank accession no. CY033001–CY033008) derived from pLLB plasmids expressing gene segments 1–7 of HK-wt to generate recombinant NS mutants. Virus rescue was performed using an 8 plasmid system as described previously [62], [63]. Briefly, DNA and Lipofectamine 2000 (Invitrogen) were mixed in a 1 ug∶2 uL ratio, incubated at room temperature for 30 minutes and then used to transfect 90% confluent 293-T cell monolayers in 6-well plates. Following 16 hour incubation, the cells were supplemented with Opti-MEM (Gibco, Carlsbad, CA) containing 1 ug/mL TPCK trypsin (Thermo Fisher Scientific, Waltham, USA). Two days later, the supernatants were used to inoculate 10-day old SPF chicken embryos for virus propagation. Virus rescue was assessed by hemagglutination assay with chicken red blood cells (Canadian Food Inspection Agency, Ottawa). To ensure absence of unwanted mutations, viral RNA was extracted from 140 ul of stock allantoic fluid from each recombinant virus using the QIAamp Viral RNA Mini Kit (Qiagen, Mississauga, Ontario) before reverse transcription and sequencing of each NS genome segment as previously described [42]. Recombinant NS mutants with NEP mutations (Fig. 1a) are indicated by an ^†^ throughout the text.

### Mouse infections

All mouse infections in this study employed 19–21 gram female CD-1 mice (Charles River Laboratories, Montreal, Quebec, Canada).

### Calculation of median lethal dose (MLD_50_)

Groups of 5–6 mice were infected intranasally under halothane anaesthesia with 50 µL volumes of 10 fold serial dilutions in PBS of viruses. Mortality and weight loss were monitored for 14 days. MLD_50_ was calculated using the Karber-Spearman method [64].

### Survival assays of recombinant HK NS mutants

Groups of 5–6 mice were infected intranasally under halothane anaesthesia with 5×10^6^ PFU of the rHK NS mutants or rHK-wt, with the exception of NS1 mutant M106V+M124I, where the infection was performed at 2.5×10^6^ PFU dose due to insufficient virus titre. Mortality and body weight loss were monitored for 14 days.

### Viral growth and IFN-β production in the mouse lung

Groups of 6–12 mice were infected intranasally under halothane anaesthesia with 1×10^5^ PFU of rHK NS mutants expressing NS1 mutations D2N^†^, L98S, M106I or M106I+L98S, as well as the rHK-wt virus in a 50 µL volume. At days 1 and 3 pi (post infection), 3–6 mice per group were euthanized by CO_2_ narcosis, lungs were extracted, suspended in 4 volumes by weight of cold sterile PBS (phosphate buffered saline) and homogenized. Virus titre was determined by plaque assay and quantified as PFU/gram (PFU/g). IFN-β concentration was determined by ELISA assay using the manufacturer's protocol (PBL biosciences, New Jersey, USA) with subtraction of mock PBS-infected lung values.

### Immunofluorescence and H &E staining of the mouse lung

Lung staining was performed as described previously [45]. Briefly, mice were infected intranasally with 1×10^5^ PFU in a 50 µL volume of the rHK NS mutants expressing NS1 mutations D2N^†^, L98S, M106I or M106I+L98S, as well as the rHK-wt virus. Lungs were collected at 2 and 6 days pi, venously perfused with PBS, and then inflated and fixed with 3.7% formaldehyde. Following 24 h fixation at 4°C, the buffer was replaced with 20% sucrose for 24 h at 4°C, and then frozen and cut into 7 uM sections. Viral antigen was detected by incubating the sections with anti-HK primary rabbit antibody diluted (1/1,000) in antibody buffer (10 mM PBS containing 3% bovine serum albumin and 0.3% Triton X-100). After washing with 10 mM PBS, the slides were incubated with Cy3-conjugated donkey anti-rabbit secondary antibody (Jackson ImmunoResearch laboratories Inc., ME) diluted (1/800) in the antibody buffer. The slides were then washed, and nuclei were stained by incubation with 100 µl Hoechst (0.2 µg/ml). Images were taken at 20× magnification using an Olympus BX50 microscope and processed in parallel manner using Adobe Photoshop version 7.0. For lung pathological examination, frozen sections were stained with hematoxylin and eosin (H&E), and examined under light microscopy for histopathologic changes. The images were obtained on a Nikon microscope using a 10× objective lens.

### *In vitro* growth of recombinant HK NS mutants

To determine the effect of MA NS1 mutations on viral replication in mouse cells, confluent M1 cells were left untreated or treated with 1000 U/mL recombinant mouse IFN-β for 24 hours, then infected at a multiplicity of infection (MOI) of 0.02 with rHK NS mutants as well as rHK-wt virus in triplicate (n = 3). Cells were infected by incubation with virus for 45 minutes at 37°C, and then supplemented with serum-free MEM (minimal essential media) in the presence of 0.5 µg/mL TPCK-trypsin (Thermo Fisher Scientific, Canada). Supernatants were collected at 12, 24, 48 and 72 hpi (hours post infection), and virus titre was determined by plaque assay in duplicate.

### Measurement of influenza viral mRNA by quantitative PCR

M1 cells were infected with rHK NS mutant or rHK- wt viruses at a MOI of 2. Forty-five minutes after incubation at 37°C, the cells were washed once with PBS and fresh serum-free MEM was added. Then, the cells were washed twice by cold PBS before collection at 8 hpi. Total RNA was isolated from infected cells by using RNeasy Protect Mini Kit (QIAGEN). 500 ng of total RNA, which corresponds to equal cell equivalents, was reverse transcribed using an oligo(dT) primer for viral mRNA. Real-time PCR (qPCR) was performed with SYBR GreenER qPCR SuperMix (Bio-Rad Laboratories (Canada) Ltd., Mississuaga, Ontario) and the Bio-Rad Chromo 4 Real-Time PCR Detector (Bio-Rad Laboratories (Canada) Ltd., Mississuaga, Ontario). Briefly, 2 µl of 10 fold diluted cDNA was added to the qPCR reaction mixture [10 µl SYBR GreenER qPCR SuperMix (2×), 1 µl each of forward and reverse gene specific primer (5 pM), 7.5 µl double-distilled water]. The cycle conditions of qPCR were 95°C 3 min, followed by 40 cycles of 95°C 30 s, 55°C 30 s and 72°C 30 s, then extended at 72°C for 3 min. Results were analyzed by ΔΔ CT methods and normalized to the β-actin gene. Each experiment was performed for three biological repeats and two technical replicates. Real-time PCR primers for A/Hong Kong/1/1968 (H3N2) NS1, NP, and M1 mRNA were matched in PCR efficiency (within ±10%) with mouse β-actin forward: AAATCGTGCGTGACATCAAA, and mouse β-actin reverse: AAGGAAGGCTGGAAAAGAGC primers, and were as follows: NP forward: TGGAGAGGTGAAAATGGACG, NP reverse: TTTCCTGGGTTCCGACTTTC NS1 forward: ACCATTGCCTTCTCTTCCAG, NS1 reverse: TCCCATTCTCATTACTGCTTCC, M1 forward: GTACGTTCTCTCTATCGTCCC, M1 reverse: GTCTTGTCTTTAGCCATTCCA.

### RNA polymerase assay

To compare the effect of mutant NS gene segments on viral polymerase activity, a luciferase reporter minigenome assay was used as per previous protocol [5]. Briefly, 96 well plates of HEK 293-T cells were transfected with 0.06 ug of phPOL1-NP-LUC reporter plasmid [5], 0.06 ug of the internal control luciferase expression plasmid PRL-SV40 (Promega), 0.06 ug of each pLLB HK-wt PB2, PB1, PA and NP, and 0.06 ug of either HK-wt or mutant pLLB NS plasmids or empty pLLB vector, using 0.5 uL lipofectamine 2000 in 100 uL Opti-MEM (Invitrogen, Burlington, Ontario). The HK-wt NS plasmid was mutated using PCR mutagenesis to express NS1 protein alone by disrupting the 3′ NS2/NEP splice site with a synonymous mutation as well as introducing a stop codon at position 14 in the NS2 reading frame using the following primers: NS1-forward: GCCTTCTCTTCCCGGACATACTATAGAGGATGTCA, and NS1 reverse: TGACATCCTCTATAGTATGTCCGGGAAGAGAAGGC. To express NS2/NEP protein alone the HK-wt NS plasmid was mutated using PCR mutagenesis to delete the NS intron using the following primers: NS2-forward: ACACTGTGTCAAGTTTTCAGGACATACTATTGAGGATGTC, and NS2 reverse: GACATCCTCAATAGTATGTCCTGAAAACTTGACACAGTGT. Forty-eight hours following transfection, luminescence was measured using the Promega Dual-Glo Luciferase Assay System with a Glomax Multi Detection System, Model 9301-010 (Fisher Scientific, Nepean, Ontario) according to the manufacturer's protocol. Data represent the average ± standard deviation of relative luciferase activities (ratio of firefly and renilla luciferase luminescence for 3 or 4 replicate experiments).

### Viral protein synthesis *in vitro*

M1 cell monolayers were treated with 200 U/ml of recombinant murine IFN-β (Sigma, St. Louis, USA) in serum-free MEM for 24 hours or left untreated. Following treatment, the monolayers were washed twice with 1× PBS then infected with the recombinant HK NS mutants as well as rHK-wt (MOI = 2) for 30 min at 37°C. Following infection, the cells were incubated with serum-free media in the absence of trypsin. At 2, 4, 6, and 8 hpi, cell monolayers were washed with PBS then the media was replaced with serum-free Met- Cys- DMEM (Gibco, Carlsbad, USA) containing 80 µCi/mL ^35^S-labeled cysteine and methionine (Express tag, Perkin Elmer, Canada) for a 1 hour pulse at 37°C, before the cell lysate was collected in 0.5 mL 1× SDS (sodium dodecyl sulphate) buffer. Samples were then used for SDS PAGE electrophoresis and autoradiography. The experiment was performed in duplicate, with representative data shown. Densitometry was performed on two independent experiments by analysis of pixel density (UNScan-it 6.1), and presented as the values relative to rHK-wt ± SE.

### Pulse chase assay

M1 cell monolayers in 12-well plates were washed twice with 1× PBS then infected with the recombinant NS mutants D2N^†^, D125G, M106V+M124I and R227K^†^ as well as rHK-wt (MOI = 2) for 30 min at 37°C. Monolayers were pulse labeled at 8 hpi, after washing with PBS, with serum-free Met- Cys- DMEM (Gibco, Carlsbad, USA) containing 80 µCi/mL ^35^S-labeled cysteine and methionine (Express tag, Perkin Elmer, Canada) for a 1 hour at 37°C. Cell lysates was either collected in 0.225 mL 1× SDS (sodium dodecyl sulphate) buffer (pulse samples) or the cells were washed twice with 1× PBS then the media was replaced with serum-free MEM supplemented with an additional 15 mg/L cold methionine and cysteine (chase samples). Chase sample cell lysates were collected at 1 and 3 hours following pulse labeling (10 and 12 hpi) in 0.225 mL 1X SDS buffer. Samples were then used for SDS PAGE electrophoresis and autoradiography. For quantification of M1 and NS1 band intensity, each gel was exposed to a PhosphorScreen (GE healthsciences) that was then read on a Typhoon Image Analyzer (GE healthsciences). Relative band intensity was quantified using Imagequant software (GE healthsciences) and calculated as the average ± standard deviation of the ratio of M1 to NS1 protein in each condition. The experiment was performed in triplicate, with representative data shown. Statistical analysis was performed, however no virus induced statistically significant (p<0.05) difference in the ratio of M1∶NS1 for pulse compared to chase.

### NS1-CPSF30 F2F3 binding assay

The CPSF30-F2F3 domain responsible for the binding of CPSF to NS1 as well as each HK-wt and mutant NS1 protein were radiolabeled and synthesized as described previously using the TnT T7 Quick-Coupled Transcription/Translation System (Promega) [65]. The F2F3 fragment was synthesized as a flag-tagged construct under control of the T7 promoter in the pET17b vector. All NS mutants and HK-wt NS expression were also expressed with the T7 promoter in the bi-directional pLLB plasmid. Briefly, 0.5 ug plasmid DNA (or empty pLLB vector) was mixed with 10 uCi ^35^S-labeled methionine+cysteine (Express ^35^S Protein Labeling Mix, Perkin Elmer) and 40 uL TnT master mix in a 50 uL total volume. The mixture was incubated 90 minutes in a 30°C water bath then stored at −20°C until use. To verify equal expression of the NS1 proteins and expression of the F2F3-FLAG fragment, an aliquot of each TnT reaction was resuspended in 3X SDS buffer, then subject to SDS-PAGE electrophoresis and autoradiography (Fig. 5b). To assess NS1 binding to F2F3-FLAG, equal volumes (10 uL of TnT reaction) of each radiolabeled NS1 (or empty pLLB vector TnT reaction) were incubated with 10 uL of radiolabeled F2F3-FLAG in the presence of Protein G Dynabeads (Invitrogen, Burlington, Ontario) pre-incubated with rabbit α-FLAG M2 (Sigma) antibody in NP-40 lysis buffer. The pulldown assay was performed as per the manufacturor's protocol of Protein G Dynabeads. Briefly, the proteins were incubated with the antibody-conjugated beads overnight at 4°C while rocking, then were washed with NP-40 lysis buffer four times using a magnetic rack. Following wash, the beads were resuspended in 50 uL 1xSDS buffer and heated at 100°C for 1 minute before analyzed by SDS-PAGE electrophoresis and autoradiography. For quantification of band intensity, each gel was exposed to a PhosphorScreen (GE healthsciences) that was then read on a Typhoon Image Analyzer (GE healthsciences). Relative band intensity was quantified using Imagequant software (GE healthsciences) for a total of three independent experiments, and calculated as the average ± standard deviation of the % value relative to HK-wt NS1. The absence of non-specific binding of the NS1 protein (wt or mutant) to the α-FLAG M2-conjugated beads was shown by pull-down in the absence of F2F3-FLAG protein (Fig. S1).

### Nucleotide sequencing

Nucleotide sequencing was done as described previously for RT-PCR amplified genome segment 8 (Ping, 2011) (Genbank accession numbers CY044265, CY044273, CY045720, HM641216, CY033125, HM641215, CY044281, CY045736, CY033533, CY033541, CY033549 and JN896299).

### Statistical analysis

Statistical significance was measured using the student t-test (Microsoft Excel, 2007) or paired student t-test (GraphPad Prism 5) using the parameters of equal variance (unless otherwise indicated) and two-tailed significance with a probability of ≤0.05 considered as statistically significant. Values were calculated as means ± standard deviation with the exception of Figure 9c–d, where values were calculated as means ± standard error from two independent experiments.

## Supporting Information

Figure S1 **Recombinant NS1 proteins do not show non-specific binding to α-FLAG M2 antibody bound Protein G Dynabeads.** NS1 proteins (wt or mutant) in the absence of CPSF30-F2F3-FLAG were not pulled-down using α-FLAG M2 antibody bound to Protein G Dynabeads relative to a control pull down in the presence of CPSF30-F2F3-FLAG (first lane). Samples were prepared and analyzed as described in methods.
(TIF)

Figure S2 **Effect of NS (NS1 +NS2/NEP) and individual NS1 and NS2/NEP proteins on RNA polymerase activity in the luciferase mini-genome assay.** Viral RNA polymerase activity was measured in HEK 293-T cells expressing HK-wt NS (NS1+NS2/NEP proteins) as well as individual NS1 or NS2/NEP proteins, or empty vector control, in combination with HK-wt PB2, PB1, PA, NP, and the NP promoter driven firefly luciferase mini-genome. Polymerase activity is shown as the ratio of firefly to renilla luciferase standardized to the vector control (100%), and presented as means ± SD for 4 experiments as well as the corresponding renilla luciferase activities (*p<0.05, **p<0.01 ***p<0.001, student's t test).
(TIF)
