# Supplementary figures and images for: Multifunctional Adaptive NS1 Mutations Are Selected upon Human Influenza Virus Evolution in the Mouse
Source: PLoS One. 2012 Feb 21;7(2):e31839. doi: 10.1371/journal.pone.0031839 (PMC3283688; doi:10.1371/journal.pone.0031839)

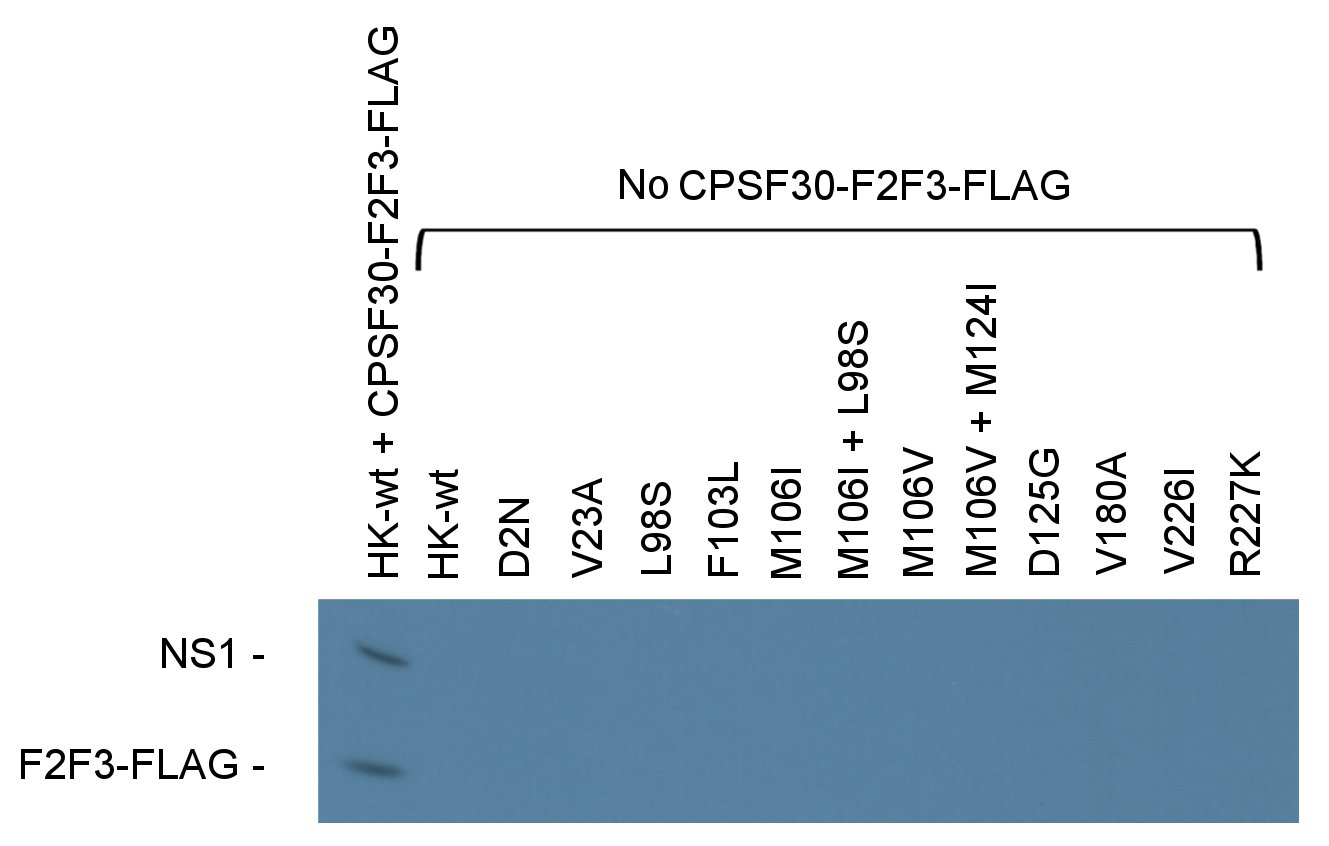

Supplement: Figure S1 — Recombinant NS1 proteins do not show non-specific binding to α-FLAG M2 antibody bound Protein G Dynabeads. NS1 proteins (wt or mutant) in the absence of CPSF30-F2F3-FLAG were not pulled-down using α-FLAG M2 antibody bound to Protein G Dynabeads relative to a control pull down in the presence of CPSF30-F2F3-FLAG (first lane). Samples were prepared and analyzed as described in methods. (TIF) [file pone.0031839.s001.tif]

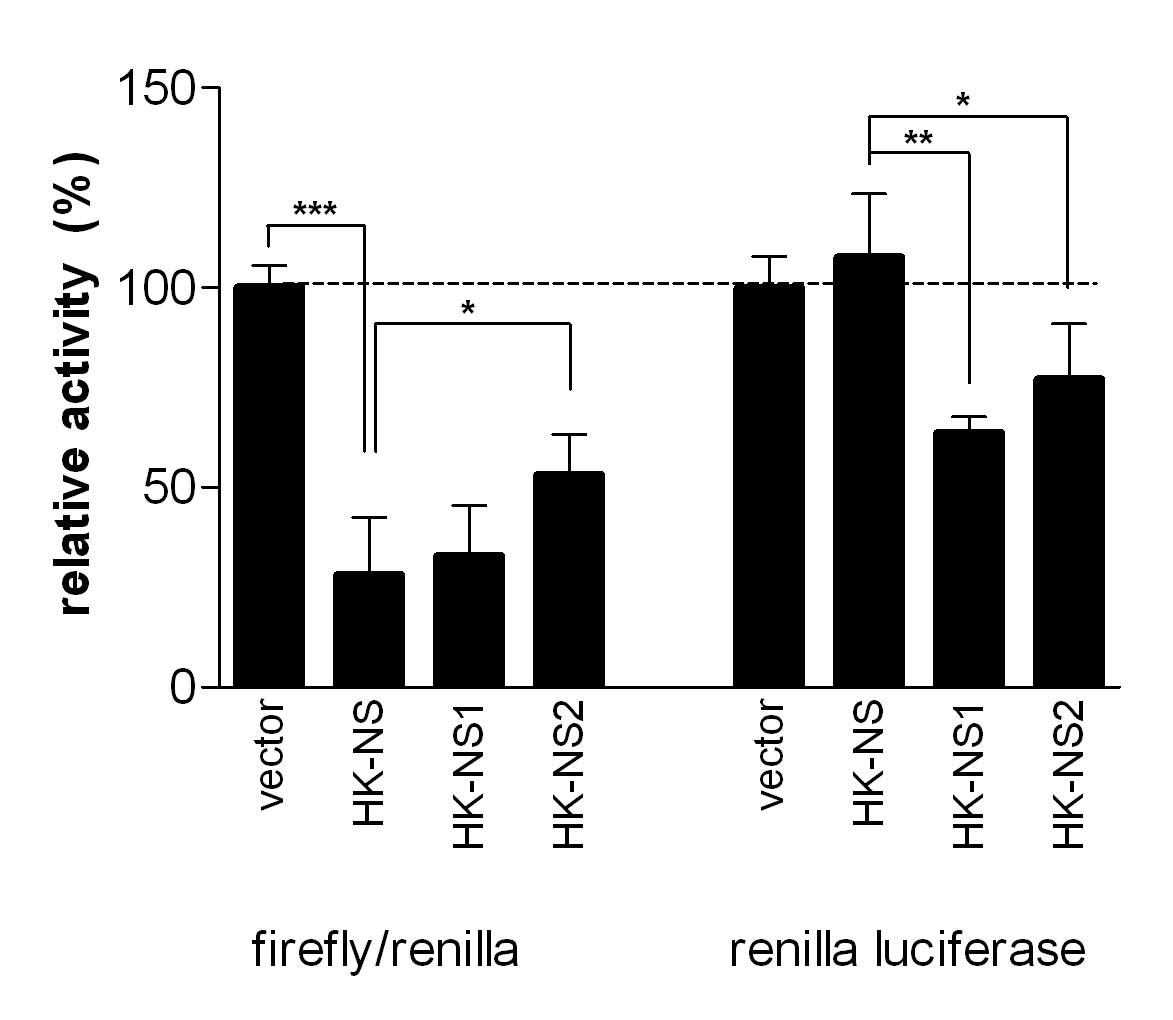

Supplement: Figure S2 — Effect of NS (NS1 +NS2/NEP) and individual NS1 and NS2/NEP proteins on RNA polymerase activity in the luciferase mini-genome assay. Viral RNA polymerase activity was measured in HEK 293-T cells expressing HK-wt NS (NS1+NS2/NEP proteins) as well as individual NS1 or NS2/NEP proteins, or empty vector control, in combination with HK-wt PB2, PB1, PA, NP, and the NP promoter driven firefly luciferase mini-genome. Polymerase activity is shown as the ratio of firefly to renilla luciferase standardized to the vector control (100%), and presented as means ± SD for 4 experiments as well as the corresponding renilla luciferase activities (*p<0.05, **p<0.01 ***p<0.001, student's t test). (TIF) [file pone.0031839.s002.tif]
